# Associations between joint effusion in the knee and gene expression levels in the circulation: a meta-analysis

**DOI:** 10.12688/f1000research.7763.1

**Published:** 2016-01-27

**Authors:** Marjolein J. Peters, Yolande F.M. Ramos, Wouter den Hollander, Dieuwke Schiphof, Albert Hofman, André G. Uitterlinden, Edwin H.G. Oei, P. Eline Slagboom, Margreet Kloppenburg, Johan L. Bloem, Sita M.A. Bierma-Zeinstra, Ingrid Meulenbelt, Joyce B.J. van Meurs

**Affiliations:** 1 Department of Internal Medicine, Erasmus MC, Rotterdam, Netherlands; 2 Department of Molecular Epidemiology, Leiden University Medical Center, Leiden, Netherlands; 3 Department of General Practice, Erasmus MC, Rotterdam, Netherlands; 4 Department of Epidemiology, Erasmus MC, Rotterdam, Netherlands; 5 Department of Radiology, Erasmus MC University Medical Center, Rotterdam, Netherlands; 6 Department of Clinical Epidemiology and Rheumatology, Leiden University Medical Center, Leiden, Netherlands; 7 Department of Radiology, Leiden University Medical Center, Leiden, Netherlands; 8 Department of Orthopedics, Erasmus MC, Rotterdam, Netherlands

**Keywords:** knee osteoarthritis, joint effusion, molecular markers, inflammation, blood

## Abstract

***Objective:*** To identify molecular biomarkers for early knee osteoarthritis (OA), we examined whether joint effusion in the knee associated with different gene expression levels in the circulation.

***Materials and Methods:*** Joint effusion grades measured with magnetic resonance (MR) imaging and gene expression levels in blood were determined in women of the Rotterdam Study (N=135) and GARP (N=98). Associations were examined using linear regression analyses, adjusted for age, fasting status, RNA quality, technical batch effects, blood cell counts, and BMI. To investigate enriched pathways and protein-protein interactions, we used the DAVID and STRING webtools.

***Results: ***In a meta-analysis, we identified 257 probes mapping to 189 unique genes in blood that were nominally significantly associated with joint effusion grades in the knee. Several compelling genes were identified such as *C1orf38* and *NFATC1*. Significantly enriched biological pathways were: response to stress, gene expression, negative regulation of intracellular signal transduction, and antigen processing and presentation of exogenous pathways.

***Conclusion:*** Meta-analyses and subsequent enriched biological pathways resulted in interesting candidate genes associated with joint effusion that require further characterization. Associations were not transcriptome-wide significant most likely due to limited power. Additional studies are required to replicate our findings in more samples, which will greatly help in understanding the pathophysiology of OA and its relation to inflammation, and may result in biomarkers urgently needed to diagnose OA at an early stage.

## Introduction

Osteoarthritis (OA) is a common, age-related, degenerative disease of the synovial joints. It is characterized by cartilage degradation, osteophyte formation, subchondral bone changes, and synovitis ^1^. These characteristics can lead to joint space narrowing, pain, and loss of function, until at the end-stage of the disease total joint replacement is required. OA is a leading cause of morbidity and disability and carries high socioeconomic costs. With increasing obesity and age in the population, a massive rise in morbidity and costs attributed to OA is expected. To be able to change from symptomatic treatment at late disease state and total joint replacement towards early (secondary) prevention, it is very important to identify new osteoarthritic disease stage markers that could be measured in the early stages of OA. These markers should function as new targets or biomarkers for early disease treatment and prevention.

Radiography is routinely used to support the diagnosis of OA. However, radiographic imaging is inadequate to detect and monitor biochemical changes within joint tissues which can occur long before symptoms are present. Magnetic resonance (MR) imaging is a non-invasive 3D imaging method with high tissue contrast that has been successfully used to visualize osteoarthritic changes ^2^. In addition to radiographic osteophyte formation and joint space loss, joint effusion can be assessed. Joint effusion is the presence of increased intra-articular fluid ^3^, which has been positively associated with knee pain in knee OA patients ^4^. Joint effusion is known to be related to joint inflammation ^5^ and a recent study showed that occurrence of joint effusion is a strong predictor for development of incident radiographic OA ^6^.

As inflammation is increasingly considered to be an important pathway in the OA pathophysiology, efforts have been made to identify pro- and anti-inflammatory mediators (such as cytokines) which enable monitoring of the OA disease course ^7– 9^. With the aim to better understand the downstream consequences of inflammation in the knee, we compared gene expression levels in the blood of participants with different grades of joint effusion, as assessed by MR imaging. Ramos *et al.* already identified specific gene expression networks in blood associated with OA status ^10^. Therefore, it could be advocated that blood expression profiles may reflect predisposition to OA. And because blood is a readily accessible tissue, gene expression levels associated with joint effusion may serve as molecular biomarkers for early detection of OA. We examined in two cohort studies whether joint effusion grades on MR imaging of the knee were associated with specific gene expression levels in the peripheral circulation, and subsequently performed a meta-analysis. Analysis for enrichment was performed to determine whether particular pathways were overrepresented among the genes associated with joint effusion.

## Materials and methods

### Subject selection

The Rotterdam Study (RS) is a large prospective, population-based cohort study in the district of Rotterdam, the Netherlands, investigating the prevalence, incidence, and risk factors of various chronic disabling diseases among elderly Caucasians aged 45 years and over. A detailed description of the design and rationale of the Rotterdam Study has been published elsewhere ^11^. We invited the first 1,116 women aged 45–60 years visiting the research center to join a sub-study investigating early signs of knee osteoarthritis (knee OA). Participants were evaluated for the self-reported presence of rheumatoid arthritis (RA) and these cases were excluded. An additional exclusion criterion was the presence of any contra-indications for MR imaging, including weighing more than 150 kilograms. In total, 891 participants were included. For this study, we selected participants having both gene expression data and good quality knee MR imaging data available. In total, we could include 135 participants. The Rotterdam Study has been approved by the Medical Ethics Committee of the Erasmus MC and by the Ministry of Health, Welfare and Sport of the Netherlands, implementing the *“Wet Bevolkingsonderzoek: ERGO (Population Studies Act: Rotterdam Study)*”. All participants provided written informed consent to participate in the study and to obtain information from their treating physicians ^11^.

The Genetics, Arthrosis and Progression study (GARP) consists of 191 sibling pairs (n=382) of white, Dutch ancestry. All participants (age range 40–78 years; mean age 60 years) are clinically and radiographically diagnosed with primary, symptomatic OA at multiple joint sites in the hand, or in at least two joints of the following locations: hand, spine (cervical or lumbar), knee, or hip ^12^. Patients with secondary OA, such as inflammatory joint disease, major developmental diseases, bone dysplasia, major local factors or metabolic diseases as hemochromatosis were excluded. Sibling pairs (n=105) with at least one subject with symptomatic hip or knee OA (but not in a radiographic end-stage) were eligible for the MR imaging sub-study ^13^; in 5 out of 210 patients no MR imaging (one due to claustrophobia, one with a large knee that did not fit into the knee coil) or an MR imaging of insufficient quality (due to motion artefacts in three patients) was available. For this study, a subset of 98 women (including 28 siblings) was selected for which both gene expression data and knee MR imaging data were available. The GARP study has been approved by the Medical Ethics Committee of the Leiden University Medical Center, the Netherlands (protocol nr. P76/98). All participants provided written informed consent to participate in the study.

### Knee OA definition

In both RS and GARP, radiographs were scored to examine knee OA. Knee OA was defined as at least one definite osteophyte and definite joint space narrowing or at least two definite osteophytes (Kellgren and Lawrence (K/L) score ≥ 2).

### MR acquisition

In RS, all participants were scanned on a 1.5 T MRI scanner (General Electric Healthcare, Milwaukee, Wisconsin, USA) with an 8-channel cardiac coil, so that two knees could be scanned at once without repositioning the subject. The protocol consisted of a sagittal fast spin echo (FSE) proton density and T2 weighted sequence (repetition time (TR) = 4,900 ms; echo time (TE) = 11/90 ms, flip angle of 90–180, slice thickness 3.2 mm, field of view 15 cm ^2^), a sagittal FSE T2 weighted sequence with frequency selective fat suppression (TR/TE = 6800/80 ms, flip angle = 90–180, slice thickness = 3.2 mm, field of view = 15 cm ^2^), a sagittal spoiled gradient echo sequence with fat suppression (TR/TE = 20.9/2.3 ms, flip angle = 35, slice thickness = 3.2 (1.6) mm, field of view = 15 cm ^2^) and a fast-imaging employing steady-state acquisition (FIESTA) sequence (TR/TE = 5.7/1.7 ms, flip angle = 35, slice thickness = 1.6 mm, field of view = 15 cm ^2^). This FIESTA sequence was acquired in the sagittal plane. Total scanning time was 27 minutes for two knees per patient.

Acquisition of MR imaging in GARP was performed using a 1.5 - T MR imaging scanner (Philips Medical Systems, Best, the Netherlands) using a 4-channel transmit/receive knee coil as described elsewhere ^13^. The following images were obtained: coronal proton density- and T2-weighted dual spin echo (SE) images (with TR = 2,200 ms; TE = 20/80 ms; 5 mm slice thickness; 0.5 mm intersection gap; 16 cm field of view; 206 × 256 acquisition matrix); sagittal proton density- and T2-weighted dual SE images (TR = 2,200 ms; TE = 20/80 ms; 4 mm slice thickness; 0.4 mm intersection gap; 16 cm field of view; 205 × 256 acquisition matrix); sagittal three-dimensional (3D) T1-weighted spoiled gradient echo (GE) frequency selective fat-suppressed images (TR =46 ms; TE =2,5 ms; flip angle 40°; 3.0 mm slice thickness; slice overlap 1.5 mm; no gap; 18 cm field of view; 205 × 256 acquisition matrix); and axial proton density- and T2-weighted turbo spin echo (TSE) fat-suppressed images (TR = 2,500 ms; TE = 7.1/40 ms; echo train length 6,2 mm slice thickness; no gap; 18 cm field of view; 205 × 256 acquisition matrix). Total acquisition time (including the initial survey sequence) was 30 min for one knee per patient. Since the original purpose of the MR imaging study in GARP was to assess progression of OA, only one knee was imaged and no images were obtained of a knee that already had a maximum K/L score of 4 ^2^.

### Semi-quantitative joint effusion scoring

In RS, a trained reader (who was blinded for any clinical, radiographic and genetic data) scored all MR images of the knees with the semi-quantitative Knee Osteoarthritis Scoring System (KOSS), described in detail elsewhere ^2^. The joint effusion grades in the tibiofemoral joint (TFJ) and the patellofemoral joint (PFJ) were scored together (grade 0–3): 0 = joint effusion absent, 1 = small joint effusion, 2 = moderate joint effusion, and 3 = massive joint effusion. The scores of the left and the right knee were summed, resulting in one grade per person ranging from 0 to 6. An experienced musculoskeletal radiologist, also blinded for any clinical, radiographic and genetic data, scored a random sample of MR images to determine the inter-observer reliability. The inter-observer reliability was moderate to good with an intra-class correlation coefficient (ICC) of 0.83.

In GARP, MR images were also scored according to KOSS ^2^ by three readers with 3, 15, and 25 years of experience in consensus, blinded to clinical, radiographic and genetic data, as described previously ^13^. Presence of joint effusion was evaluated on T2-weigthed coronal, sagittal and axial sequences. A small, physiological sliver of synovial fluid was not recorded. A small effusion (grade 1) was present when a small amount of fluid distended one or two of the joint recesses, moderate effusion (grade 2) when more than two recesses were partially distended, and massive (grade 3) when there was full distension of all the joint recesses. As in RS, the grades were scored semi-quantitatively ranging from 0 to 3.

Because we used non-contrast-enhanced MR imaging in both GARP and RS, we could not measure synovial thickness reliably.

### Gene expression levels

In RS, whole-blood was collected (PAXGene Tubes – Becton Dickinson) and total RNA was isolated (PAXGene Blood RNA kits - Qiagen). To ensure a constant high quality of the RNA preparations, all RNA samples were analyzed using the Labchip GX (Calliper) according to the manufacturer’s instructions. Samples with an RNA Quality Score > 7 were amplified and labelled (Ambion TotalPrep RNA), and hybridized to the Illumina HumanHT12v4 Expression Beadchips. Processing of the Rotterdam Study RNA samples was performed at the Genetic Laboratory of Internal Medicine, Erasmus University Medical Center Rotterdam, and the dataset has been deposited in the GEO database under the accession number GSE33828 ^14^.

For GARP, generation of gene expression levels in peripheral blood mononuclear cells (PBMCs) has been described elsewhere ^10^. Gene expression data has been deposited in the GEO database under the accession number GSE48556.

Both RS and GARP samples were scanned on the Illumina iScan System (combined with an AutoLoader) using Illumina iScan image data acquisition software (version 3). Illumina GenomeStudio software (version 1.9.0) was used to generate output files for further statistical analyses. To identify transcripts that had detectable quantitative expression, we used the detection p-values reported by Illumina’s GenomeStudio software. The detection p-value represents the confidence that a given transcript is expressed above the background defined by negative control probes. We called a transcript significantly expressed when the detection p-value was <0.05 in more than 50 percent of all samples. All other transcripts were excluded from analysis. Because of this stringent detection p-value cut-off, the overall false-positive rate is very small (we won’t get false positive genes), whereas the false-negative rate might be higher (so we could lose some joint effusion associated genes, *i.e.,* genes that are expressed at high joint effusion grades specifically).

### Statistical- and functional analysis

Statistical analyses were performed in R (version 3.1.2) ^15^. Raw gene expression intensities were normalized by quantile-normalization to the median distribution; gene expression levels were subsequently log2-transformed. To minimize the influence of the overall signal levels, which may reflect RNA quantity and quality rather than a true biological difference between individuals, the probe means and sample means were centered to zero, and sample variance was linearly scaled, such that each sample had a standard deviation of one (standardization). To identify transcripts that were differentially expressed with joint effusion grades, we used four different linear regression models (lm):

- *Model 0: unadjusted: lm (probe ~ joint effusion grade)*
- *Model 1: adjusted for age + fasting status + RNA quality score (RQS) + batch + cell counts*
- *Model 2: adjusted for Model 1 + body mass index (BMI)*
- *Model 3: adjusted for Model 1 + BMI + nonsteroidal anti-inflammatory drug (NSAID) intake*

BMI was measured at the research centers (as weight in kg divided by height ^2^ in meters), and NSAID intake was extracted from the pharmacy records (RS) or collected via questionnaires (GARP). Because it is known that BMI is associated with markers of inflammation ^16, 17^, and because additional adjustments for NSAID use (model 3) hardly changed the effect sizes and standard errors of the results as shown in the Supplementary Table 1– Supplementary Table 2, we used model 2 for the meta-analysis and follow-up analyses. Notably, the analysis in GARP was also adjusted for siblingship in addition to age, batch, and BMI. In GARP, no adjustments were included for fasting status since blood was collected for all participants without fasting. Furthermore, gene expression levels were assessed from PBMCs and the RNA integrity number (or RQS) was at least 8.3 (36 random samples were analyzed) ^10^.

To be able to meta-analyze the results of both studies, we combined the 12,843 Illumina HT12v4 probes (RS) and the 12,246 Illumina HT12v3 probes (GARP) based on chromosomal position and nucleotide sequence: 9,507 probes (representing 7,408 unique genes) were similar between the two gene expression platforms and could be meta-analyzed.

We ran sample size weighted meta-analyses based on p-values and the direction of the effects. By using the p-values and the effect direction, a Z-statistic characterizing the evidence for association was calculated. The Z-statistic summarized the magnitude and the direction of the effect. An overall Z-statistic and p-value was calculated from the weighted sum of the individual statistics. Weights were proportional to the square-root of the number of individuals examined in each sample and standardized such that the squared weights sum to 1. We used the Meta-Analysis Tool (version: generic-metal-2011-03-25) for genome-wide association scans (METAL) ^18^ for this. METAL has been developed for meta-analyzing genetic genome-wide association studies. Because we are dealing with gene expression levels and not SNPs, we changed the SNPID column to probe IDs and assigned all probes a minor allele A and a major allele G, a minor allele frequency = 0.10, and a + strand. For the positions, the probe chromosomes and the midpoint position of the probes were used. Sample sizes, effect directions, and p-values were extracted from the linear regression model results files. Probes with a meta-analysis p-value<6.75E-06 (0.05/7,408 genes tested) were considered transcriptome-wide significantly associated with the joint effusion grades in the knee.

### Pathway analyses

Pathway analysis was done with the DAVID tool; the *Database for annotation, visualization and integrated discovery* (version 6) ^19^. We included all nominal significant genes (meta-analysis p-value <0.05), and checked for enrichment of any biological processes identified in the gene ontology database.

### Analysis of protein interaction networks

To investigate protein interactions among the nominal significant genes, we used the *Search Tool for the Retrieval of Interacting Genes/Proteins* (version 9.1) ^20^, which is available online. With the “enrichment” option, we checked for enrichment of protein-protein interactions and “ *GO biological processes*”.

## Results

### Subjects

**Figure 1.**
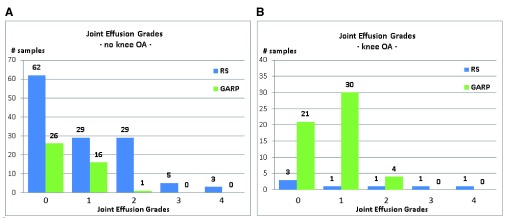
Joint effusion grades in subjects without knee OA ( **A**) and samples with knee OA ( **B**).

The complete characteristics of the included subjects of both RS and GARP are shown in Table 1 and Figure 1. In both, RS and GARP, mean age of the subjects with and without joint effusion was not significantly different (ANOVA p-value RS = 0.146, ANOVA p-value GARP=0.181). Mean BMI seemed to be higher with higher joint effusion grades, but due to small sample sizes this difference was not significant (ANOVA p-value RS = 0.069, ANOVA p-value GARP=0.487).

**Table 1.** Subject characteristics of RS and GARP. * *this can be in one or two knees.*

	RS				GARP			
joint effusion grades	#	Mean Age (+/- SD)	Mean BMI (+/- SD)	# knee OA*	#	Mean Age (+/- SD)	Mean BMI (+/- SD)	# knee OA*
Grade 0	65	54.0 (3.4)	26.6 (4.6)	3	47	60.7 (6.9)	26.5 (3.8)	21
Grade 1	30	55.1 (3.9)	28.0 (5.3)	1	46	58.9 (6.6)	25.8 (3.9)	30
Grade 2	30	54.8 (3.8)	26.9 (4.4)	1	5	58.3 (8.9)	27.2 (7.3)	4
Grade 3	6	56.2 (2.1)	29.8 (4.9)	1	0	-	-	-
Grade 4	4	52.0 (4.2)	36.8 (10.1)	1	0	-	-	-
Grade 5	0	-	-	-	0	-	-	-
Grade 6	0	-	-	-	0	-	-	-
**Total:**	**135**	**54.5 (3.6)**	**27.4 (5.2)**	**7**	**98**	**59.7 (6.8)**	**26.1 (4.0)**	**55**

### Results within RS

Of the 7,408 genes tested, *CLEC4A* (C-type lectin domain family 4, member A) demonstrated the strongest association with joint effusion grades in the knee (effect size=0.407 (SE=0.120); p-value =9.57E-04). In total, 310 probes (representing 251 unique genes) were nominally significant. The top 50 results are shown in Supplementary Table 1.

### Results within GARP

In GARP, the lowest p-value was found for the DNA-damage-inducible transcript 4 ( *DDIT4*) gene (effect size=-1.425 (SE=0.411); p-value=5.21E-04). In total, 439 probes (representing 331 unique genes) were nominally significant ( Supplementary Table 2).

### Meta-analysis of RS and GARP

In general, the top five genes of GARP and RS were different. To identify a common transcriptional signature for joint effusion, we performed a meta-analysis across RS and GARP. The top 20 results are shown in Table 2. All 257 nominally significant probes (representing 189 unique genes) are shown in Supplementary Table 3. The lowest p-value was found for the *C1orf38* (Chromosome 1 Open Reading Frame 38) gene, also called *THEMIS2* (Thymocyte Selection Associated Family Member 2) or *ICB-1* (Induced by Contact to Basement membrane) (Zscore=-3.356; p-value=7.90E-04). Gene expression levels of *C1orf38* were lower in samples with higher joint effusion grades in both whole blood and PBMCs ( Supplementary Figure 1). Also the *DYNLL2* gene (Dynein, Light Chain, LC8-Type 2), the *NFATC1* gene (Nuclear factor of activated T-cells, cytoplasmic 1), and the *RBM4* gene (RNA Binding Motif Protein 4) were nominally associated, with respectively higher ( *DYNLL2* and *NFATC1*) and lower ( *RBM4*) gene expression levels correlating with advanced joint effusion grades ( Supplementary Figure 2– Supplementary Figure 4).

**Table 2.** Top 20 results of the meta-analysis (n=257).

*Gene*	*ILMN ID*	*RS*			*GARP*			*META-ANALYSIS*			*RS* *position*	*GARP* *position*
		*Effect*	*SE*	*P-value*	*Effect*	*SE*	*P-value*	*Zscore*	*P-value*	*Dir*
***C1orf38***	2470240	-0.132	0.056	2.00E-02	-0.170	0.121	1.63E-01	-3.356	7.90E-04	--	56	93
***GABPB1***	7200431	-0.108	0.053	4.35E-02	-0.190	0.094	4.30E-02	-3.325	8.84E-04	--	185	27
***TMEM97***	3420541	-0.118	0.053	2.65E-02	-0.304	0.126	1.61E-02	-3.099	1.94E-03	--	95	167
***DYNLL2***	3400551	0.090	0.053	8.91E-02	0.101	0.103	3.27E-01	3.087	2.02E-03	++	425	33
***RBM4***	510132	-0.081	0.054	1.36E-01	-0.285	0.093	2.10E-03	-3.067	2.16E-03	--	739	15
***PRICKLE1***	1770224	0.124	0.053	2.03E-02	0.400	0.177	2.41E-02	2.993	2.76E-03	++	54	429
***AP3B1***	2230603	0.155	0.073	3.61E-02	0.203	0.103	4.80E-02	2.989	2.80E-03	++	133	195
***TUBB2C***	2070368	-0.062	0.068	3.60E-01	-0.349	0.102	5.98E-04	-2.922	3.48E-03	--	2634	2
***FKBP14***	6100411	0.141	0.052	7.69E-03	0.157	0.089	7.90E-02	2.915	3.56E-03	++	19	1499
***GFM1\LXN***	60670	0.108	0.066	1.05E-01	0.346	0.124	5.29E-03	2.899	3.74E-03	++	521	78
***LYZ***	4810162	-0.561	0.204	6.95E-03	-0.338	0.553	5.42E-01	-2.832	4.62E-03	--	15	2031
***ARL6IP1***	2690047	0.146	0.070	3.98E-02	0.189	0.112	9.07E-02	2.831	4.64E-03	++	148	359
***PTPLB***	6980253	0.091	0.067	1.79E-01	0.359	0.110	1.13E-03	2.809	4.96E-03	++	1098	32
***MED19***	3450427	-0.099	0.059	9.51E-02	-0.084	0.116	4.66E-01	-2.79	5.28E-03	--	478	117
***APTX***	1570138	-0.081	0.040	4.37E-02	-0.118	0.083	1.57E-01	-2.783	5.39E-03	--	174	371
***NFATC1***	940725	0.112	0.061	7.11E-02	0.216	0.120	7.15E-02	2.778	5.46E-03	++	319	194
***RG9MTD3\*** ***SHB***	1770196	0.147	0.051	5.04E-03	0.174	0.098	7.65E-02	2.755	5.87E-03	++	10	3222
***POLR2J***	6350333	-0.154	0.056	7.14E-03	-0.445	0.152	3.46E-03	-2.74	6.14E-03	--	20	2330
***-***	1070754	0.170	0.056	3.14E-03	-0.038	0.074	6.05E-01	2.737	6.20E-03	++	7	4195
***IFT43***	3170458	-0.130	0.043	2.99E-03	-0.152	0.081	6.15E-02	-2.734	6.26E-03	--	8	4228

### Pathway-analysis of genes nominally significant in the meta-analysis

Of the 189 unique genes represented by the 257 nominally associated probes (p-value<0.05), 178 genes were recognized by the webtool DAVID. The most significant GO terms identified were: *intracellular protein transport* (GO:0006886: 13 of 374 genes, p-value=4.5E-04, Fold Enrichment (FE)=3.4), *response to stress* (GO:0006950: 34 of 1685 genes, p-value=1.5E-04, FE=2.0), *antigen processing and presentation of exogenous antigens* (GO:0019884: 4 of 14 genes, p-value=3.5E-04, FE=27.8), but the three GO terms did not survive the Benjamini Hochberg multiple testing correction. Additionally, one KEGG pathway was nominally significantly enriched: *antigen processing and presentation* (hsa04612: 5 of 83 genes, p-value=0.0127, FE=5.4).

**Figure 2.**
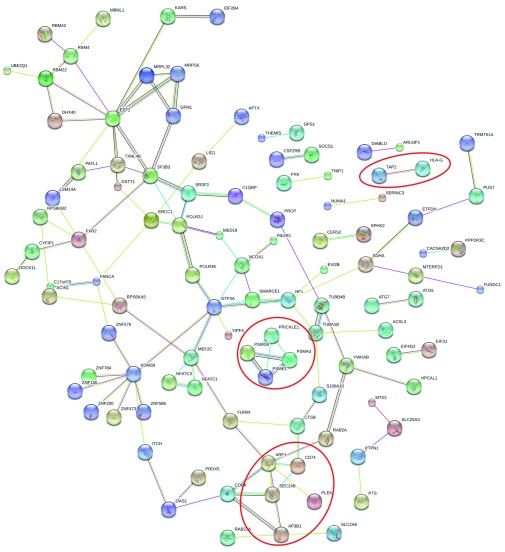
Protein-protein interactions determined with STRING showing interactions between the 178 nominally associated genes (p-value<0.05), marking proteins involved in antigen processing and presentation of exogenous antigens in red (GO:0019884). Disconnected proteins are hidden.

Using the webtool STRING, we did not find significantly enriched protein-protein interactions within the network of 178 genes (p-value=0.386, observed interactions=58, expected interactions=55). However, STRING confirmed two significantly enriched biological pathways identified with DAVID: *response to stress* (45 of 1685 genes, p-value=6.23E-03) and *antigen processing and presentation of exogenous antigens* (10 of 14 genes, p-value=3.44E-02). The protein-protein interactions are visualized in Figure 2. Proteins involved in the antigen processing and presentation of exogenous antigens pathway (GO:0019884) are marked red, highlighting a cluster of three proteasomes (PSMA3, PSMD6, and PSME1) important for the antigen processing pathway.

## Discussion

We examined whether joint effusion grades in the knee were associated with specific gene expression levels in the circulation, which could potentially serve as molecular biomarker to indicate OA in the early stage. We identified 257 nominally associated probes (p-value<0.05) mapping to 189 unique genes. *C1orf38*, *DYNLL2*, and *RBM4* were among the 5 most significant genes in the meta-analysis. Additional adjustments for BMI and NSAID intake did not notably affect the results, suggesting that the associations are consistent across all BMI ranges and in both users and non-users of NSAIDs. Subsequent pathway analyses with DAVID revealed nominal significant enrichment of genes involved in response to stress, gene expression, negative regulation of intracellular signal transduction, and antigen processing and presentation of exogenous antigens pathways. The biological pathways response to stress and antigen processing and presentation of exogenous antigens were confirmed with a second pathway analysis tool STRING.

*C1orf38* is a protein-coding gene and is highly expressed in several blood cells (monocytes, dendritic cells, NK-cells, T-cells, B-cells). The gene is induced by interferon-gamma (IFN-γ), an important cytokine that orchestrates many distinct cellular processes regarding inflammation ^21^. Therefore, *C1orf38* could be an interesting candidate for further research.

Cytoplasmic dynein consists of a molecular complex of several proteins including *DYNLL2*, and it is thought to play a role in movement and positioning of a wide range of organelles and complexes in the cell ^22^. Notably, recent studies showed that *DYNLL2* inhibits inflammation and may also inhibit osteoclastogenesis and bone resorption via regulation of *NFκB* transcription activity ^23^. This would suggest that the higher expression of *DYNLL2* in association with higher joint effusion grades is rather consequence than cause, however, this remains to be established.

*RBM4* is thought to play a role in alternative splice site selection during pre-mRNA processing, and seems to be important for the regulation of the translation of pro-inflammatory genes ^24^.

Of note is the association of higher joint effusion grades with higher expression levels of *NFATC1* (nuclear factor of activated T cells 1). Besides its function in bone remodeling through calcium/calcineurin signaling, *NFATC1* belongs to a family of transcription factors that play a central role in inducible gene transcription during immune response ^25^. Although no significant differences were found in *NFATC1* gene expression between OA-affected and unaffected tissues using microarray analyses ^26– 29^, a slight but significant reduction was detected by RT-qPCR in OA affected cartilage ^30^. In addition, Jeffries and colleagues ^31^ found changes in DNA methylation profiles, and it was shown that cartilage-specific ablation of *NFATC1* predisposes to development of early onset OA too ^30^. Since the expression of *NFATC1* is positively associated with joint effusion it could be speculated that, in line with the increased expression of *DYNLL2*, upon occurrence of joint effusion specific pathways are activated to protect against development of OA. Consistent with this hypothesis, we observed that increased expression of *NFATC1* in association with joint effusion is much more pronounced in subjects without knee OA in GARP. Therefore, *NFATC1* might be a useful biomarker for early detection of OA. However, this should be confirmed in a longitudinal study tracking the development of the disease.

The pathway enrichment analysis results were consistent with known inflammatory disease mechanisms including response to stress and gene expression. Cellular stress and inflammation are known to reciprocally activate or inhibit each other, depending on the immune cell type and the stress-inducing signals ^32^. Additionally, we identified the pathways negative regulation of intracellular signal transduction (GO:1902532) and antigen processing and presentation of exogenous antigens (GO:0019884). Hanada *et al.* ^33^ already highlighted a key role for the intracellular signal transduction pathways of the pro- and anti-inflammatory cytokines which activate inflammatory transcription factors such as *NF-κB*, *Smad*, and *STATs*. The antigen processing machinery can be easily linked to the inflammatory response too ^34^.

STRING showed the interaction between 3 proteasomes identified in the analysis (PSMA3, PSMD6, and PSME1). Proteasomes are important for degrading intracellular proteins, and recently it has been shown that mutations and polymorphisms in the proteasome are associated with several inflammatory and auto-inflammatory diseases ^35^. Therefore, these genes could also be interesting targets for future studies.

Despite the identification of several compelling potential markers for early OA, a major drawback of the current study was the relatively small sample size (n=233). Although gene expression data and knee MR images are available in larger datasets, the number of samples in which both measurements were determined is unfortunately limited. In addition, the data of the two cohorts (RS and GARP) was rather heterogeneous in particular due to the fact that in RS joint effusion grades were combined for two knees (sum of left and right knee), while in GARP joint effusion was determined in one randomly selected knee. Moreover, GARP is a cohort of clinical OA cases while RS is a population-based cohort study, in which no selection was made for OA cases specifically: in RS only seven out of 135 subjects (5.2%) were diagnosed with radiographically evident knee osteoarthritis, while in GARP 55 out of 98 subjects (56.1%) had knee OA. Furthermore, in RS the analyses were adjusted for fasting status (134 of 135 subjects fasted overnight) and RNA quality scores, while in GARP non-fasting subjects were used and RNA quality scores were available in a small subset only. Finally, gene expression levels in RS were determined in whole blood, while in GARP PBMCs were used. Although a previous study showed that expression levels differ across different RNA sources (whole blood, PBMCs, and lymphoblastoid cell lines), phenotype-based differential expression analyses results were consistent in whole blood and PBMCs ^36^. Taken together, it is likely that cohort heterogeneity has resulted in limited power due to which no transcriptome-wide significant probes were identified. Earlier studies confirmed the good quality and reproducibility of our gene expression arrays ^10, 27, 37^. Another potential limitation of our study is that we did not assess recent traumatic knee injuries: traumas can increase joint effusion and dilute our associations.

In conclusion, joint effusion grades in the knee on MR imaging were nominally associated with the expression levels of 189 unique genes in blood and the identified genes were mainly involved in inflammation. Although the associations presented in this manuscript were not transcriptome-wide significant, the meta-analysis and subsequent enriched biological pathways resulted in compelling candidate genes such as *C1orf38* and *NFATC1* that could be further characterized in future research. Additional studies are needed to replicate our findings as well as to identify other genes which will greatly help in understanding the pathophysiology of OA and its relation with inflammation, and may result in biomarkers urgently needed to diagnose OA at an early stage.

## Supplementary Material

Supplementary figures and tables for ‘Associations between joint effusion in the knee and gene expression levels in the circulation: a meta-analysis’.
