# Supplementary material for: Associations between joint effusion in the knee and gene expression levels in the circulation: a meta-analysis
Source: F1000Res. 2016 Jan 27;5:109. [Version 1] doi: 10.12688/f1000research.7763.1 (PMC4837985; doi:10.12688/f1000research.7763.1)
Supplement: Supplementary file 1 [file f1000research-5-8357-s0000.tgz › ecd588b8-8829-4331-90aa-223a08d28642.docx]

**SUPPLEMENTAL TABLES**

**Supplementary Table 1: Top 50 results of the Rotterdam Study (n=135 samples)**

| ***Gene*** | ***ILMN ID*** | ***Model 0:  unadjusted*** | | | ***Model 1: adj age + technical covariates*** | | | ***Model 2:  adj Model 1 + BMI*** | | | ***Model 3:  adj Model 2 + NSAID use*** | | |
| --- | --- | --- | --- | --- | --- | --- | --- | --- | --- | --- | --- | --- | --- |
|  |  | *Effect* | *SE* | *Pvalue* | *Effect* | *SE* | *Pvalue* | *Effect* | *SE* | *Pvalue* | *Effect* | *SE* | *Pvalue* |
| ***CLEC4A*** | 4050202 | 0.362 | 0.135 | 8.22E-03 | 0.486 | 0.119 | 8.50E-05 | 0.407 | 0.120 | 9.57E-04 | 0.403 | 0.120 | 1.08E-03 |
| ***OR2W5*** | 7330605 | -0.189 | 0.066 | 4.72E-03 | -0.194 | 0.062 | 2.35E-03 | -0.214 | 0.064 | 1.20E-03 | -0.216 | 0.064 | 1.07E-03 |
| ***MMAB*** | 2140291 | -0.121 | 0.054 | 2.62E-02 | -0.152 | 0.054 | 5.89E-03 | -0.179 | 0.055 | 1.56E-03 | -0.174 | 0.055 | 1.87E-03 |
| ***TESK1*** | 4860315 | 0.193 | 0.071 | 7.82E-03 | 0.206 | 0.065 | 1.93E-03 | 0.217 | 0.067 | 1.60E-03 | 0.210 | 0.066 | 1.76E-03 |
| ***RBM42*** | 4880551 | 0.253 | 0.097 | 9.80E-03 | 0.181 | 0.063 | 4.59E-03 | 0.199 | 0.064 | 2.52E-03 | 0.204 | 0.064 | 1.80E-03 |
| ***PTPRO*** | 1660386 | -0.137 | 0.050 | 6.43E-03 | -0.146 | 0.048 | 2.91E-03 | -0.150 | 0.049 | 3.02E-03 | -0.155 | 0.048 | 1.78E-03 |
| ***CAP1*** | 1070754 | 0.222 | 0.084 | 9.41E-03 | 0.168 | 0.058 | 4.78E-03 | 0.182 | 0.060 | 3.13E-03 | 0.178 | 0.060 | 3.52E-03 |
| ***IFT43*** | 3170458 | -0.122 | 0.048 | 1.24E-02 | -0.134 | 0.044 | 2.82E-03 | -0.138 | 0.046 | 3.14E-03 | -0.132 | 0.044 | 3.52E-03 |
| ***-*** | 7210184 | 0.124 | 0.053 | 2.13E-02 | 0.122 | 0.053 | 2.25E-02 | 0.157 | 0.053 | 3.70E-03 | 0.160 | 0.053 | 3.22E-03 |
| ***TRMT10B*** | 1770196 | 0.128 | 0.051 | 1.35E-02 | 0.161 | 0.052 | 2.47E-03 | 0.156 | 0.054 | 4.57E-03 | 0.156 | 0.054 | 5.05E-03 |
| ***C8orf33*** | 6350671 | 0.171 | 0.053 | 1.68E-03 | 0.146 | 0.051 | 5.26E-03 | 0.152 | 0.053 | 4.83E-03 | 0.154 | 0.053 | 4.31E-03 |
| ***NIN*** | 1740114 | -0.158 | 0.058 | 7.43E-03 | -0.165 | 0.057 | 4.43E-03 | -0.168 | 0.059 | 5.02E-03 | -0.162 | 0.058 | 5.79E-03 |
| ***RRBP1*** | 4540327 | 0.110 | 0.054 | 4.27E-02 | 0.128 | 0.052 | 1.43E-02 | 0.149 | 0.053 | 5.77E-03 | 0.151 | 0.053 | 5.35E-03 |
| ***TMEM194A*** | 1110494 | -0.141 | 0.057 | 1.46E-02 | -0.174 | 0.056 | 2.61E-03 | -0.163 | 0.059 | 6.41E-03 | -0.167 | 0.057 | 4.18E-03 |
| ***LYZ*** | 4810162 | -0.532 | 0.219 | 1.64E-02 | -0.567 | 0.210 | 7.94E-03 | -0.601 | 0.218 | 6.75E-03 | -0.599 | 0.220 | 7.48E-03 |
| ***NFXL1*** | 1230731 | 0.268 | 0.162 | 1.00E-01 | 0.401 | 0.144 | 6.32E-03 | 0.411 | 0.150 | 7.03E-03 | 0.400 | 0.149 | 8.19E-03 |
| ***ATP6V1D*** | 1660736 | 0.198 | 0.099 | 4.84E-02 | 0.254 | 0.079 | 1.81E-03 | 0.220 | 0.080 | 7.19E-03 | 0.220 | 0.081 | 7.79E-03 |
| ***COMMD4*** | 2370093 | 0.232 | 0.109 | 3.50E-02 | 0.235 | 0.084 | 5.89E-03 | 0.235 | 0.086 | 7.23E-03 | 0.241 | 0.086 | 5.99E-03 |
| ***FKBP14*** | 6100411 | 0.132 | 0.054 | 1.50E-02 | 0.14 | 0.053 | 1.01E-02 | 0.151 | 0.055 | 7.27E-03 | 0.151 | 0.055 | 7.60E-03 |
| ***POLR2J*** | 6350333 | -0.158 | 0.058 | 7.43E-03 | -0.158 | 0.058 | 7.48E-03 | -0.162 | 0.060 | 7.99E-03 | -0.165 | 0.060 | 7.03E-03 |
| ***SAMD3*** | 1030167 | 0.470 | 0.147 | 1.74E-03 | 0.339 | 0.117 | 4.37E-03 | 0.326 | 0.121 | 8.14E-03 | 0.316 | 0.119 | 9.22E-03 |
| ***HPCAL1*** | 520184 | -0.192 | 0.091 | 3.68E-02 | -0.237 | 0.080 | 3.85E-03 | -0.222 | 0.083 | 8.81E-03 | -0.223 | 0.084 | 9.02E-03 |
| ***CHTF18*** | 1770689 | -0.149 | 0.048 | 2.18E-03 | -0.138 | 0.048 | 5.06E-03 | -0.134 | 0.050 | 8.97E-03 | -0.134 | 0.051 | 9.10E-03 |
| ***IL8*** | 1570553 | -0.293 | 0.109 | 7.87E-03 | -0.283 | 0.101 | 6.06E-03 | -0.276 | 0.104 | 9.27E-03 | -0.283 | 0.104 | 7.50E-03 |
| ***SPARC*** | 5050382 | 0.166 | 0.062 | 8.81E-03 | 0.171 | 0.061 | 6.22E-03 | 0.169 | 0.064 | 9.39E-03 | 0.166 | 0.064 | 1.09E-02 |
| ***-*** | 3390681 | -0.167 | 0.075 | 2.70E-02 | -0.154 | 0.075 | 4.25E-02 | -0.198 | 0.076 | 1.06E-02 | -0.196 | 0.077 | 1.20E-02 |
| ***NREP*** | 940471 | 0.172 | 0.077 | 2.66E-02 | 0.187 | 0.072 | 1.07E-02 | 0.194 | 0.075 | 1.07E-02 | 0.191 | 0.075 | 1.23E-02 |
| ***PSMD1*** | 5090647 | 0.185 | 0.073 | 1.22E-02 | 0.131 | 0.052 | 1.39E-02 | 0.140 | 0.054 | 1.11E-02 | 0.139 | 0.054 | 1.15E-02 |
| ***ACPL2*** | 3800538 | 0.141 | 0.059 | 1.74E-02 | 0.147 | 0.059 | 1.40E-02 | 0.158 | 0.061 | 1.11E-02 | 0.159 | 0.061 | 1.09E-02 |
| ***CERK*** | 2230367 | 0.171 | 0.075 | 2.49E-02 | 0.164 | 0.065 | 1.25E-02 | 0.173 | 0.067 | 1.13E-02 | 0.170 | 0.067 | 1.31E-02 |
| ***RUFY1*** | 2030079 | 0.135 | 0.064 | 3.71E-02 | 0.17 | 0.059 | 4.91E-03 | 0.158 | 0.062 | 1.16E-02 | 0.161 | 0.062 | 1.04E-02 |
| ***CACNA2D2*** | 3120180 | 0.148 | 0.056 | 8.87E-03 | 0.129 | 0.054 | 1.93E-02 | 0.143 | 0.056 | 1.21E-02 | 0.142 | 0.056 | 1.33E-02 |
| ***CLIC3*** | 5870136 | 0.426 | 0.182 | 2.06E-02 | 0.393 | 0.161 | 1.64E-02 | 0.425 | 0.167 | 1.22E-02 | 0.426 | 0.167 | 1.20E-02 |
| ***INIP*** | 990475 | 0.106 | 0.061 | 8.20E-02 | 0.156 | 0.060 | 1.07E-02 | 0.157 | 0.062 | 1.31E-02 | 0.158 | 0.063 | 1.37E-02 |
| ***-*** | 1440519 | 0.132 | 0.060 | 2.88E-02 | 0.161 | 0.062 | 1.08E-02 | 0.162 | 0.065 | 1.35E-02 | 0.165 | 0.065 | 1.28E-02 |
| ***PIH1D1*** | 5910202 | -0.136 | 0.077 | 7.81E-02 | -0.162 | 0.066 | 1.50E-02 | -0.170 | 0.068 | 1.35E-02 | -0.166 | 0.068 | 1.55E-02 |
| ***PIN4*** | 1030475 | 0.084 | 0.044 | 6.08E-02 | 0.092 | 0.044 | 3.89E-02 | 0.113 | 0.045 | 1.41E-02 | 0.114 | 0.046 | 1.35E-02 |
| ***FAM49A*** | 2510184 | 0.086 | 0.077 | 2.68E-01 | 0.152 | 0.067 | 2.41E-02 | 0.171 | 0.069 | 1.45E-02 | 0.167 | 0.066 | 1.34E-02 |
| ***APTX*** | 6590674 | 0.111 | 0.045 | 1.55E-02 | 0.135 | 0.044 | 3.05E-03 | 0.112 | 0.045 | 1.49E-02 | 0.109 | 0.045 | 1.69E-02 |
| ***ARPC2*** | 7160327 | 0.138 | 0.076 | 7.22E-02 | 0.174 | 0.062 | 5.99E-03 | 0.157 | 0.064 | 1.61E-02 | 0.156 | 0.065 | 1.77E-02 |
| ***FAM105A*** | 5130661 | -0.102 | 0.063 | 1.10E-01 | -0.143 | 0.056 | 1.21E-02 | -0.141 | 0.058 | 1.67E-02 | -0.144 | 0.058 | 1.44E-02 |
| ***-*** | 3370037 | 0.065 | 0.106 | 5.42E-01 | 0.136 | 0.074 | 6.88E-02 | 0.181 | 0.075 | 1.75E-02 | 0.176 | 0.073 | 1.78E-02 |
| ***MRPS6*** | 6400195 | 0.174 | 0.074 | 2.11E-02 | 0.158 | 0.067 | 2.00E-02 | 0.168 | 0.070 | 1.76E-02 | 0.167 | 0.070 | 1.94E-02 |
| ***-*** | 5340519 | 0.144 | 0.066 | 3.12E-02 | 0.150 | 0.069 | 3.05E-02 | 0.170 | 0.071 | 1.76E-02 | 0.171 | 0.071 | 1.75E-02 |
| ***-*** | 6480246 | -0.116 | 0.077 | 1.33E-01 | -0.163 | 0.068 | 1.83E-02 | -0.170 | 0.071 | 1.77E-02 | -0.162 | 0.068 | 1.97E-02 |
| ***METTL3*** | 6860524 | -0.057 | 0.069 | 4.12E-01 | -0.132 | 0.054 | 1.72E-02 | -0.136 | 0.057 | 1.80E-02 | -0.138 | 0.057 | 1.64E-02 |
| ***YWHAB*** | 1340521 | 0.093 | 0.085 | 2.77E-01 | 0.098 | 0.067 | 1.49E-01 | 0.159 | 0.066 | 1.81E-02 | 0.155 | 0.066 | 2.03E-02 |
| ***COX19*** | 1450255 | -0.208 | 0.076 | 7.32E-03 | -0.202 | 0.066 | 2.88E-03 | -0.159 | 0.067 | 1.90E-02 | -0.153 | 0.066 | 2.21E-02 |
| ***FIS1*** | 6420242 | -0.070 | 0.136 | 6.10E-01 | -0.130 | 0.123 | 2.90E-01 | -0.274 | 0.115 | 1.90E-02 | -0.274 | 0.116 | 2.00E-02 |
| ***EMG1*** | 4850091 | 0.159 | 0.069 | 2.19E-02 | 0.190 | 0.067 | 5.67E-03 | 0.164 | 0.069 | 1.93E-02 | 0.161 | 0.069 | 2.20E-02 |

**Supplementary Table 2: Top 50 results of GARP (n=98 samples)**

| ***Gene*** | ***ILMN ID*** | ***Model 0:  unadjusted*** | | | ***Model 1: adj age + technical covariates*** | | | ***Model 2:  adj Model 1 + BMI*** | | | ***Model 3:  adj Model 2 + NSAID use*** | | |
| --- | --- | --- | --- | --- | --- | --- | --- | --- | --- | --- | --- | --- | --- |
|  |  | *Effect* | *SE* | *Pvalue* | *Effect* | *SE* | *Pvalue* | *Effect* | *SE* | *Pvalue* | *Effect* | *SE* | *Pvalue* |
| ***DDIT4*** | 3190148 | -0.991 | 0.361 | 6.11E-03 | -1.415 | 0.416 | 6.70E-04 | -1.425 | 0.411 | 5.21E-04 | -1.425 | 0.414 | 5.76E-04 |
| ***TUBB4B*** | 2070368 | -0.612 | 0.143 | 1.75E-05 | -0.437 | 0.128 | 6.24E-04 | -0.439 | 0.127 | 5.63E-04 | -0.437 | 0.128 | 6.34E-04 |
| ***MUTYH*** | 5670037 | -0.270 | 0.107 | 1.19E-02 | -0.362 | 0.105 | 5.56E-04 | -0.361 | 0.106 | 6.38E-04 | -0.358 | 0.107 | 8.15E-04 |
| ***MBIP*** | 6480041 | 0.255 | 0.104 | 1.47E-02 | 0.319 | 0.097 | 1.06E-03 | 0.320 | 0.098 | 1.09E-03 | 0.315 | 0.098 | 1.26E-03 |
| ***KLF4*** | 2810059 | 0.472 | 0.251 | 5.98E-02 | 0.768 | 0.240 | 1.34E-03 | 0.780 | 0.240 | 1.14E-03 | 0.766 | 0.242 | 1.58E-03 |
| ***DEPDC5*** | 1090291 | -0.252 | 0.095 | 8.03E-03 | -0.279 | 0.089 | 1.73E-03 | -0.274 | 0.088 | 1.89E-03 | -0.274 | 0.089 | 2.03E-03 |
| ***CSHL1*** | 1690102 | 0.254 | 0.116 | 2.91E-02 | 0.269 | 0.086 | 1.79E-03 | 0.262 | 0.084 | 1.91E-03 | 0.259 | 0.085 | 2.32E-03 |
| ***DET1*** | 6650452 | -0.219 | 0.097 | 2.43E-02 | -0.305 | 0.099 | 1.99E-03 | -0.306 | 0.099 | 2.04E-03 | -0.306 | 0.100 | 2.23E-03 |
| ***SMARCD1*** | 3400593 | 0.289 | 0.116 | 1.32E-02 | 0.404 | 0.133 | 2.32E-03 | 0.395 | 0.129 | 2.16E-03 | 0.393 | 0.130 | 2.41E-03 |
| ***PATL1*** | 5310427 | 0.324 | 0.123 | 8.57E-03 | 0.329 | 0.108 | 2.35E-03 | 0.329 | 0.109 | 2.48E-03 | 0.331 | 0.109 | 2.36E-03 |
| ***EIF4G2*** | 2260095 | 0.089 | 0.255 | 7.28E-01 | 0.427 | 0.147 | 3.56E-03 | 0.438 | 0.145 | 2.53E-03 | 0.449 | 0.145 | 1.98E-03 |
| ***PRDX5*** | 520750 | -0.348 | 0.131 | 8.07E-03 | -0.347 | 0.115 | 2.48E-03 | -0.346 | 0.115 | 2.66E-03 | -0.353 | 0.115 | 2.14E-03 |
| ***ING3*** | 7550707 | 0.352 | 0.169 | 3.71E-02 | 0.455 | 0.151 | 2.65E-03 | 0.456 | 0.152 | 2.69E-03 | 0.465 | 0.154 | 2.51E-03 |
| ***SLC41A3*** | 940373 | -0.288 | 0.122 | 1.82E-02 | -0.340 | 0.113 | 2.59E-03 | -0.341 | 0.114 | 2.75E-03 | -0.336 | 0.116 | 3.71E-03 |
| ***-*** | 510132 | -0.355 | 0.099 | 3.44E-04 | -0.341 | 0.111 | 2.14E-03 | -0.332 | 0.111 | 2.81E-03 | -0.336 | 0.110 | 2.37E-03 |
| ***CLPX*** | 6760050 | 0.486 | 0.139 | 4.73E-04 | 0.425 | 0.142 | 2.80E-03 | 0.426 | 0.143 | 2.86E-03 | 0.425 | 0.144 | 3.10E-03 |
| ***RBP7*** | 3850112 | -0.672 | 0.218 | 2.06E-03 | -0.650 | 0.216 | 2.62E-03 | -0.649 | 0.218 | 2.87E-03 | -0.649 | 0.220 | 3.19E-03 |
| ***C11orf73*** | 780440 | -0.353 | 0.100 | 4.40E-04 | -0.337 | 0.116 | 3.69E-03 | -0.341 | 0.115 | 3.09E-03 | -0.339 | 0.117 | 3.60E-03 |
| ***-*** | 3130358 | -0.275 | 0.123 | 2.61E-02 | -0.354 | 0.119 | 3.05E-03 | -0.353 | 0.120 | 3.31E-03 | -0.357 | 0.121 | 3.18E-03 |
| ***ATF6*** | 5420343 | -0.326 | 0.122 | 7.79E-03 | -0.394 | 0.136 | 3.71E-03 | -0.392 | 0.137 | 4.10E-03 | -0.392 | 0.138 | 4.42E-03 |
| ***ZNF200*** | 7100300 | -0.447 | 0.173 | 9.88E-03 | -0.552 | 0.191 | 3.92E-03 | -0.552 | 0.193 | 4.20E-03 | -0.566 | 0.190 | 2.91E-03 |
| ***ULK3*** | 4480132 | -0.253 | 0.128 | 4.86E-02 | -0.355 | 0.123 | 4.02E-03 | -0.354 | 0.124 | 4.35E-03 | -0.353 | 0.125 | 4.67E-03 |
| ***PFKFB3*** | 1470601 | -0.217 | 0.192 | 2.59E-01 | -0.393 | 0.138 | 4.46E-03 | -0.395 | 0.139 | 4.40E-03 | -0.392 | 0.139 | 4.86E-03 |
| ***SLMO2*** | 7000403 | 0.615 | 0.183 | 7.58E-04 | 0.418 | 0.146 | 4.28E-03 | 0.419 | 0.147 | 4.50E-03 | 0.411 | 0.146 | 4.92E-03 |
| ***ARPC5*** | 20142 | -0.527 | 0.226 | 1.98E-02 | -0.421 | 0.149 | 4.72E-03 | -0.424 | 0.149 | 4.52E-03 | -0.408 | 0.150 | 6.38E-03 |
| ***ENTPD4*** | 2900224 | -0.252 | 0.168 | 1.34E-01 | -0.469 | 0.167 | 5.06E-03 | -0.470 | 0.168 | 5.03E-03 | -0.490 | 0.170 | 3.93E-03 |
| ***GABPB1*** | 7200431 | -0.201 | 0.099 | 4.15E-02 | -0.305 | 0.111 | 6.21E-03 | -0.309 | 0.111 | 5.11E-03 | -0.321 | 0.109 | 3.23E-03 |
| ***-*** | 4570576 | 0.581 | 0.143 | 4.97E-05 | 0.398 | 0.142 | 4.93E-03 | 0.398 | 0.143 | 5.27E-03 | 0.399 | 0.143 | 5.25E-03 |
| ***PANK4*** | 7000553 | -0.331 | 0.127 | 9.33E-03 | -0.334 | 0.122 | 6.15E-03 | -0.339 | 0.122 | 5.33E-03 | -0.346 | 0.122 | 4.59E-03 |
| ***RAB11FIP1*** | 6130156 | -0.263 | 0.127 | 3.85E-02 | -0.395 | 0.142 | 5.42E-03 | -0.391 | 0.140 | 5.34E-03 | -0.391 | 0.141 | 5.69E-03 |
| ***EMC9*** | 4570014 | -0.429 | 0.157 | 6.11E-03 | -0.352 | 0.128 | 5.84E-03 | -0.356 | 0.128 | 5.41E-03 | -0.363 | 0.128 | 4.65E-03 |
| ***PTPLB*** | 6980253 | 0.138 | 0.193 | 4.74E-01 | 0.351 | 0.135 | 8.98E-03 | 0.364 | 0.131 | 5.62E-03 | 0.363 | 0.132 | 5.95E-03 |
| ***DYNLL2*** | 3400551 | 0.243 | 0.149 | 1.02E-01 | 0.324 | 0.121 | 7.31E-03 | 0.329 | 0.120 | 5.91E-03 | 0.341 | 0.119 | 4.30E-03 |
| ***ABCF1*** | 7380113 | -0.217 | 0.120 | 7.04E-02 | -0.301 | 0.108 | 5.28E-03 | -0.297 | 0.108 | 6.05E-03 | -0.292 | 0.108 | 6.97E-03 |
| ***EMC6*** | 3990600 | -0.619 | 0.175 | 4.10E-04 | -0.403 | 0.149 | 6.95E-03 | -0.406 | 0.148 | 6.06E-03 | -0.416 | 0.147 | 4.64E-03 |
| ***CSRNP2*** | 1110372 | -0.174 | 0.090 | 5.32E-02 | -0.277 | 0.100 | 5.77E-03 | -0.276 | 0.101 | 6.16E-03 | -0.274 | 0.101 | 6.98E-03 |
| ***PPHLN1*** | 2810730 | 0.317 | 0.120 | 8.27E-03 | 0.357 | 0.130 | 6.20E-03 | 0.358 | 0.131 | 6.21E-03 | 0.358 | 0.132 | 6.70E-03 |
| ***TNFRSF10B*** | 2600463 | -0.341 | 0.144 | 1.79E-02 | -0.380 | 0.140 | 6.55E-03 | -0.384 | 0.140 | 6.24E-03 | -0.364 | 0.137 | 7.79E-03 |
| ***ME2*** | 7550521 | 0.377 | 0.114 | 9.41E-04 | 0.327 | 0.118 | 5.58E-03 | 0.323 | 0.118 | 6.30E-03 | 0.324 | 0.118 | 6.16E-03 |
| ***CXCR4*** | 1300280 | -0.579 | 0.226 | 1.06E-02 | -0.713 | 0.262 | 6.44E-03 | -0.709 | 0.260 | 6.31E-03 | -0.711 | 0.261 | 6.47E-03 |
| ***HSD17B11*** | 6580487 | 0.265 | 0.147 | 7.11E-02 | 0.349 | 0.142 | 1.37E-02 | 0.364 | 0.133 | 6.37E-03 | 0.358 | 0.134 | 7.37E-03 |
| ***MED8*** | 3060619 | -0.345 | 0.112 | 2.14E-03 | -0.350 | 0.127 | 5.79E-03 | -0.348 | 0.127 | 6.38E-03 | -0.351 | 0.127 | 5.72E-03 |
| ***ZNF160*** | 360709 | 0.348 | 0.118 | 3.15E-03 | 0.339 | 0.124 | 6.09E-03 | 0.339 | 0.124 | 6.42E-03 | 0.335 | 0.125 | 7.51E-03 |
| ***MRPL32*** | 4480327 | -0.343 | 0.129 | 7.84E-03 | -0.352 | 0.133 | 8.27E-03 | -0.358 | 0.132 | 6.56E-03 | -0.362 | 0.133 | 6.31E-03 |
| ***-*** | 1430255 | 0.345 | 0.159 | 3.02E-02 | 0.195 | 0.072 | 6.62E-03 | 0.196 | 0.072 | 6.74E-03 | 0.194 | 0.073 | 7.85E-03 |
| ***LRRC25*** | 5310397 | -0.767 | 0.326 | 1.87E-02 | -0.950 | 0.349 | 6.44E-03 | -0.941 | 0.348 | 6.92E-03 | -0.935 | 0.350 | 7.62E-03 |
| ***EGR1*** | 870338 | 2.473 | 0.879 | 4.87E-03 | 2.721 | 1.009 | 7.02E-03 | 2.717 | 1.013 | 7.35E-03 | 2.739 | 1.016 | 7.02E-03 |
| ***DDX39A*** | 4860673 | -0.461 | 0.147 | 1.69E-03 | -0.400 | 0.154 | 9.70E-03 | -0.407 | 0.152 | 7.37E-03 | -0.405 | 0.153 | 8.10E-03 |
| ***TRIM24*** | 6510196 | 0.355 | 0.115 | 1.96E-03 | 0.262 | 0.099 | 8.15E-03 | 0.265 | 0.099 | 7.41E-03 | 0.269 | 0.099 | 6.52E-03 |
| ***RPS6KB2*** | 2350538 | -0.353 | 0.114 | 1.97E-03 | -0.266 | 0.106 | 1.24E-02 | -0.287 | 0.107 | 7.58E-03 | -0.288 | 0.108 | 7.91E-03 |

**Supplementary Table 3: Results (p<0.05) of the differential expression meta-analysis (n=233 samples)**

| ***Gene*** | ***ILMN ID*** | ***RS*** | | | ***GARP*** | | | ***META-ANALYSIS*** | | | ***RS position*** | ***GARP position*** |
| --- | --- | --- | --- | --- | --- | --- | --- | --- | --- | --- | --- | --- |
|  |  | *Effect* | *SE* | *Pvalue* | *Effect* | *SE* | *Pvalue* | *Zscore* | *Pvalue* | *Dir* |  |  |
| ***C1orf38*** | 2470240 | -0.132 | 0.056 | 2.00E-02 | -0.170 | 0.121 | 1.63E-01 | -3.356 | 7.90E-04 | -- | 56 | 93 |
| ***GABPB1*** | 7200431 | -0.108 | 0.053 | 4.35E-02 | -0.190 | 0.094 | 4.30E-02 | -3.325 | 8.84E-04 | -- | 185 | 27 |
| ***TMEM97*** | 3420541 | -0.118 | 0.053 | 2.65E-02 | -0.304 | 0.126 | 1.61E-02 | -3.099 | 1.94E-03 | -- | 95 | 167 |
| ***DYNLL2*** | 3400551 | 0.090 | 0.053 | 8.91E-02 | 0.101 | 0.103 | 3.27E-01 | 3.087 | 2.02E-03 | ++ | 425 | 33 |
| ***RBM4*** | 510132 | -0.081 | 0.054 | 1.36E-01 | -0.285 | 0.093 | 2.10E-03 | -3.067 | 2.16E-03 | -- | 739 | 15 |
| ***PRICKLE1*** | 1770224 | 0.124 | 0.053 | 2.03E-02 | 0.400 | 0.177 | 2.41E-02 | 2.993 | 2.76E-03 | ++ | 54 | 429 |
| ***AP3B1*** | 2230603 | 0.155 | 0.073 | 3.61E-02 | 0.203 | 0.103 | 4.80E-02 | 2.989 | 2.80E-03 | ++ | 133 | 195 |
| ***TUBB2C*** | 2070368 | -0.062 | 0.068 | 3.60E-01 | -0.349 | 0.102 | 5.98E-04 | -2.922 | 3.48E-03 | -- | 2634 | 2 |
| ***FKBP14*** | 6100411 | 0.141 | 0.052 | 7.69E-03 | 0.157 | 0.089 | 7.90E-02 | 2.915 | 3.56E-03 | ++ | 19 | 1499 |
| ***GFM1\LXN*** | 60670 | 0.108 | 0.066 | 1.05E-01 | 0.346 | 0.124 | 5.29E-03 | 2.899 | 3.74E-03 | ++ | 521 | 78 |
| ***-*** | 4810162 | -0.561 | 0.204 | 6.95E-03 | -0.338 | 0.553 | 5.42E-01 | -2.832 | 4.62E-03 | -- | 15 | 2031 |
| ***ARL6IP1*** | 2690047 | 0.146 | 0.070 | 3.98E-02 | 0.189 | 0.112 | 9.07E-02 | 2.831 | 4.64E-03 | ++ | 148 | 359 |
| ***PTPLB*** | 6980253 | 0.091 | 0.067 | 1.79E-01 | 0.359 | 0.110 | 1.13E-03 | 2.809 | 4.96E-03 | ++ | 1098 | 32 |
| ***MED19*** | 3450427 | -0.099 | 0.059 | 9.51E-02 | -0.084 | 0.116 | 4.66E-01 | -2.79 | 5.28E-03 | -- | 478 | 117 |
| ***APTX*** | 1570138 | -0.081 | 0.040 | 4.37E-02 | -0.118 | 0.083 | 1.57E-01 | -2.783 | 5.39E-03 | -- | 174 | 371 |
| ***NFATC1*** | 940725 | 0.112 | 0.061 | 7.11E-02 | 0.216 | 0.120 | 7.15E-02 | 2.778 | 5.46E-03 | ++ | 319 | 194 |
| ***RG9MTD3\SHB*** | 1770196 | 0.147 | 0.051 | 5.04E-03 | 0.174 | 0.098 | 7.65E-02 | 2.755 | 5.87E-03 | ++ | 10 | 3222 |
| ***POLR2J*** | 6350333 | -0.154 | 0.056 | 7.14E-03 | -0.445 | 0.152 | 3.46E-03 | -2.74 | 6.14E-03 | -- | 20 | 2330 |
| ***-*** | 1070754 | 0.170 | 0.056 | 3.14E-03 | -0.038 | 0.074 | 6.05E-01 | 2.737 | 6.20E-03 | ++ | 7 | 4195 |
| ***-*** | 3170458 | -0.130 | 0.043 | 2.99E-03 | -0.152 | 0.081 | 6.15E-02 | -2.734 | 6.26E-03 | -- | 8 | 4228 |
| ***C3orf31*** | 7000746 | 0.127 | 0.070 | 7.23E-02 | 0.066 | 0.122 | 5.88E-01 | 2.731 | 6.31E-03 | ++ | 331 | 221 |
| ***-*** | 3370037 | 0.169 | 0.070 | 1.80E-02 | 0.419 | 0.121 | 5.43E-04 | 2.731 | 6.32E-03 | ++ | 42 | 1289 |
| ***TXNL4B*** | 5220731 | -0.085 | 0.050 | 9.19E-02 | -0.210 | 0.095 | 2.66E-02 | -2.73 | 6.34E-03 | -- | 467 | 155 |
| ***YWHAB*** | 1340521 | 0.149 | 0.062 | 1.80E-02 | 0.243 | 0.105 | 2.11E-02 | 2.719 | 6.55E-03 | ++ | 47 | 1291 |
| ***ING2*** | 7160025 | 0.069 | 0.051 | 1.78E-01 | 0.105 | 0.084 | 2.11E-01 | 2.711 | 6.71E-03 | ++ | 1013 | 65 |
| ***-*** | 5870228 | -0.124 | 0.059 | 3.77E-02 | -0.206 | 0.079 | 9.33E-03 | -2.705 | 6.82E-03 | -- | 138 | 653 |
| ***COPA*** | 4150500 | 0.096 | 0.065 | 1.44E-01 | 0.276 | 0.079 | 4.94E-04 | 2.699 | 6.96E-03 | ++ | 784 | 95 |
| ***CAB39L*** | 780500 | 0.100 | 0.045 | 2.73E-02 | 0.124 | 0.083 | 1.35E-01 | 2.696 | 7.01E-03 | ++ | 72 | 1071 |
| ***-*** | 1440519 | 0.152 | 0.061 | 1.40E-02 | -0.054 | 0.076 | 4.78E-01 | 2.693 | 7.07E-03 | ++ | 35 | 1791 |
| ***N6AMT2*** | 3450739 | -0.088 | 0.045 | 5.52E-02 | -0.153 | 0.105 | 1.44E-01 | -2.678 | 7.40E-03 | -- | 246 | 380 |
| ***-*** | 1980669 | 0.098 | 0.048 | 4.36E-02 | 0.172 | 0.148 | 2.43E-01 | 2.678 | 7.41E-03 | ++ | 146 | 692 |
| ***-*** | 6860524 | -0.128 | 0.053 | 1.76E-02 | -0.066 | 0.124 | 5.92E-01 | -2.67 | 7.58E-03 | -- | 46 | 1506 |
| ***NDUFB4*** | 7210184 | 0.146 | 0.050 | 3.90E-03 | 0.235 | 0.220 | 2.87E-01 | 2.669 | 7.62E-03 | ++ | 9 | 4440 |
| ***EIF4G2*** | 2260095 | 0.062 | 0.067 | 3.51E-01 | 0.367 | 0.128 | 4.04E-03 | 2.668 | 7.62E-03 | ++ | 2459 | 11 |
| ***LIG1*** | 5050113 | -0.116 | 0.058 | 4.62E-02 | -0.407 | 0.119 | 6.60E-04 | -2.656 | 7.91E-03 | -- | 184 | 592 |
| ***-*** | 2320189 | 0.079 | 0.054 | 1.52E-01 | 0.238 | 0.085 | 5.20E-03 | 2.645 | 8.17E-03 | ++ | 853 | 104 |
| ***DHX40*** | 5220433 | 0.095 | 0.068 | 1.62E-01 | 0.271 | 0.116 | 1.94E-02 | 2.644 | 8.18E-03 | ++ | 900 | 99 |
| ***TMEM194A*** | 1110494 | -0.153 | 0.055 | 6.15E-03 | -0.254 | 0.106 | 1.60E-02 | -2.638 | 8.33E-03 | -- | 14 | 3499 |
| ***TATDN2*** | 2690753 | -0.071 | 0.058 | 2.24E-01 | -0.320 | 0.109 | 3.31E-03 | -2.636 | 8.38E-03 | -- | 1391 | 59 |
| ***ERCC1*** | 3610474 | 0.098 | 0.047 | 4.15E-02 | 0.250 | 0.127 | 4.94E-02 | 2.636 | 8.40E-03 | ++ | 151 | 801 |
| ***LASS2*** | 6380154 | -0.145 | 0.066 | 3.10E-02 | -0.278 | 0.115 | 1.56E-02 | -2.627 | 8.63E-03 | -- | 111 | 1020 |
| ***CD99L2*** | 1780079 | -0.101 | 0.051 | 4.78E-02 | -0.118 | 0.129 | 3.62E-01 | -2.611 | 9.04E-03 | -- | 188 | 690 |
| ***GSTT1*** | 7400537 | -0.183 | 0.116 | 1.17E-01 | -0.570 | 0.404 | 1.58E-01 | -2.587 | 9.69E-03 | -- | 628 | 190 |
| ***PIH1D1*** | 5910202 | -0.161 | 0.064 | 1.31E-02 | -0.202 | 0.084 | 1.66E-02 | -2.581 | 9.85E-03 | -- | 36 | 2473 |
| ***-*** | 610543 | 0.133 | 0.062 | 3.46E-02 | 0.175 | 0.103 | 9.14E-02 | 2.566 | 1.03E-02 | ++ | 119 | 1158 |
| ***-*** | 1690348 | -0.117 | 0.057 | 4.01E-02 | -0.182 | 0.091 | 4.53E-02 | -2.56 | 1.05E-02 | -- | 152 | 1024 |
| ***PSMD6*** | 5810070 | 0.111 | 0.048 | 2.33E-02 | 0.089 | 0.104 | 3.89E-01 | 2.542 | 1.10E-02 | ++ | 70 | 1757 |
| ***PATL1*** | 5310427 | 0.056 | 0.074 | 4.49E-01 | 0.177 | 0.088 | 4.48E-02 | 2.539 | 1.11E-02 | ++ | 3413 | 10 |
| ***SFXN1*** | 4730411 | -0.122 | 0.052 | 2.10E-02 | -0.177 | 0.155 | 2.54E-01 | -2.536 | 1.12E-02 | -- | 61 | 1977 |
| ***PIN4*** | 1030475 | 0.106 | 0.043 | 1.51E-02 | 0.158 | 0.083 | 5.70E-02 | 2.531 | 1.14E-02 | ++ | 37 | 2731 |
| ***SPARC*** | 5050382 | 0.158 | 0.060 | 1.03E-02 | 0.365 | 0.227 | 1.07E-01 | 2.529 | 1.14E-02 | ++ | 25 | 3598 |
| ***SAMD3*** | 1030167 | 0.306 | 0.114 | 8.03E-03 | 0.241 | 0.233 | 3.01E-01 | 2.512 | 1.20E-02 | ++ | 21 | 4116 |
| ***KIAA1967*** | 5050047 | -0.105 | 0.069 | 1.29E-01 | -0.322 | 0.142 | 2.38E-02 | -2.511 | 1.21E-02 | -- | 673 | 239 |
| ***-*** | 1660653 | -0.121 | 0.061 | 4.79E-02 | -0.142 | 0.100 | 1.54E-01 | -2.51 | 1.21E-02 | -- | 195 | 961 |
| ***EIF2B4*** | 6370494 | -0.054 | 0.048 | 2.61E-01 | -0.130 | 0.069 | 5.98E-02 | -2.508 | 1.21E-02 | -- | 1702 | 77 |
| ***ZNF200*** | 7100300 | -0.061 | 0.069 | 3.83E-01 | -0.439 | 0.163 | 6.97E-03 | -2.507 | 1.22E-02 | -- | 2879 | 21 |
| ***C1orf56*** | 4150332 | -0.133 | 0.058 | 2.29E-02 | 0.077 | 0.118 | 5.16E-01 | -2.488 | 1.29E-02 | -- | 77 | 1992 |
| ***RPS6KB2*** | 2350538 | -0.070 | 0.073 | 3.39E-01 | -0.327 | 0.097 | 7.08E-04 | -2.466 | 1.37E-02 | -- | 2315 | 50 |
| ***MUTYH*** | 5670037 | -0.019 | 0.057 | 7.35E-01 | -0.228 | 0.087 | 9.25E-03 | -2.462 | 1.38E-02 | -- | 6527 | 3 |
| ***ASB3*** | 2690068 | 0.121 | 0.056 | 3.26E-02 | 0.160 | 0.087 | 6.80E-02 | 2.446 | 1.45E-02 | ++ | 113 | 1761 |
| ***RAB2*** | 5360379 | 0.139 | 0.060 | 2.32E-02 | 0.145 | 0.097 | 1.35E-01 | 2.441 | 1.46E-02 | ++ | 65 | 2468 |
| ***-*** | 1410400 | 0.228 | 0.120 | 5.92E-02 | 0.566 | 0.197 | 4.09E-03 | 2.424 | 1.54E-02 | ++ | 249 | 1029 |
| ***-*** | 1470601 | -0.053 | 0.070 | 4.54E-01 | -0.262 | 0.116 | 2.39E-02 | -2.421 | 1.55E-02 | -- | 3424 | 23 |
| ***SAMD3*** | 290750 | 0.248 | 0.105 | 1.96E-02 | 0.218 | 0.224 | 3.31E-01 | 2.416 | 1.57E-02 | ++ | 53 | 2886 |
| ***HPCAL1*** | 520184 | -0.207 | 0.078 | 8.94E-03 | -0.099 | 0.099 | 3.19E-01 | -2.403 | 1.63E-02 | -- | 22 | 4927 |
| ***FUNDC1*** | 4260192 | 0.068 | 0.055 | 2.18E-01 | 0.238 | 0.095 | 1.27E-02 | 2.403 | 1.63E-02 | ++ | 1337 | 150 |
| ***RBM42*** | 4880551 | 0.186 | 0.061 | 2.62E-03 | -0.060 | 0.129 | 6.44E-01 | 2.398 | 1.65E-02 | ++ | 5 | 8354 |
| ***-*** | 4480220 | 0.067 | 0.053 | 2.12E-01 | 0.270 | 0.120 | 2.39E-02 | 2.386 | 1.70E-02 | ++ | 1228 | 182 |
| ***-*** | 6900402 | -0.088 | 0.056 | 1.21E-01 | -0.234 | 0.102 | 2.17E-02 | -2.382 | 1.72E-02 | -- | 644 | 463 |
| ***ZNF160*** | 360709 | 0.043 | 0.055 | 4.32E-01 | 0.165 | 0.100 | 9.95E-02 | 2.38 | 1.73E-02 | ++ | 3121 | 43 |
| ***SLC2A8*** | 5870326 | -0.071 | 0.065 | 2.77E-01 | -0.262 | 0.129 | 4.17E-02 | -2.369 | 1.79E-02 | -- | 1817 | 109 |
| ***-*** | 7000403 | 0.039 | 0.057 | 4.96E-01 | 0.192 | 0.118 | 1.05E-01 | 2.367 | 1.80E-02 | ++ | 3795 | 24 |
| ***PTS*** | 6280341 | -0.052 | 0.049 | 2.92E-01 | -0.171 | 0.080 | 3.35E-02 | -2.353 | 1.86E-02 | -- | 1934 | 107 |
| ***FANCA\ZNF276*** | 3190138 | -0.078 | 0.061 | 2.05E-01 | -0.314 | 0.122 | 1.00E-02 | -2.352 | 1.87E-02 | -- | 1340 | 186 |
| ***GRIPAP1*** | 540678 | -0.064 | 0.064 | 3.17E-01 | -0.204 | 0.112 | 6.84E-02 | -2.349 | 1.88E-02 | -- | 2120 | 96 |
| ***MRPL32*** | 4480327 | -0.054 | 0.069 | 4.36E-01 | -0.312 | 0.105 | 3.01E-03 | -2.348 | 1.89E-02 | -- | 3332 | 44 |
| ***ABHD2*** | 2970201 | -0.069 | 0.056 | 2.22E-01 | -0.157 | 0.088 | 7.65E-02 | -2.347 | 1.89E-02 | -- | 1481 | 160 |
| ***TEX10*** | 6770044 | -0.060 | 0.054 | 2.70E-01 | -0.173 | 0.070 | 1.37E-02 | -2.344 | 1.91E-02 | -- | 1928 | 110 |
| ***ATG7*** | 5220754 | -0.070 | 0.059 | 2.41E-01 | -0.200 | 0.124 | 1.07E-01 | -2.338 | 1.94E-02 | -- | 1553 | 156 |
| ***DHRS8*** | 6580487 | 0.065 | 0.089 | 4.64E-01 | 0.311 | 0.115 | 6.88E-03 | 2.338 | 1.94E-02 | ++ | 3472 | 41 |
| ***BCAP29*** | 6250564 | 0.073 | 0.051 | 1.55E-01 | 0.202 | 0.119 | 8.93E-02 | 2.335 | 1.95E-02 | ++ | 855 | 383 |
| ***ING4*** | 1430767 | 0.118 | 0.056 | 3.77E-02 | -0.048 | 0.098 | 6.26E-01 | 2.33 | 1.98E-02 | ++ | 125 | 2279 |
| ***-*** | 6960181 | -0.124 | 0.073 | 8.92E-02 | -0.071 | 0.061 | 2.44E-01 | -2.328 | 1.99E-02 | -- | 455 | 884 |
| ***POLR3K*** | 7000703 | -0.103 | 0.051 | 4.62E-02 | -0.009 | 0.113 | 9.40E-01 | -2.327 | 2.00E-02 | -- | 202 | 1666 |
| ***-*** | 1070358 | -0.089 | 0.052 | 9.10E-02 | -0.233 | 0.158 | 1.42E-01 | -2.324 | 2.02E-02 | -- | 444 | 900 |
| ***-*** | 60041 | -0.068 | 0.053 | 2.02E-01 | -0.264 | 0.100 | 8.35E-03 | -2.323 | 2.02E-02 | -- | 1316 | 218 |
| ***ZNF187*** | 5390259 | -0.085 | 0.046 | 6.86E-02 | -0.036 | 0.102 | 7.22E-01 | -2.322 | 2.02E-02 | -- | 320 | 1186 |
| ***SOCS1*** | 1990300 | -0.083 | 0.060 | 1.74E-01 | -0.289 | 0.170 | 8.86E-02 | -2.317 | 2.05E-02 | -- | 1012 | 328 |
| ***C1QBP*** | 7200392 | -0.145 | 0.065 | 2.80E-02 | -0.160 | 0.105 | 1.26E-01 | -2.314 | 2.07E-02 | -- | 91 | 2907 |
| ***SDHA*** | 3180470 | 0.116 | 0.061 | 5.78E-02 | 0.079 | 0.105 | 4.53E-01 | 2.314 | 2.07E-02 | ++ | 241 | 1488 |
| ***PYHIN1*** | 7000270 | 0.191 | 0.097 | 5.16E-02 | 0.229 | 0.174 | 1.88E-01 | 2.298 | 2.16E-02 | ++ | 208 | 1783 |
| ***CTSB*** | 240309 | 0.181 | 0.114 | 1.14E-01 | 0.606 | 0.188 | 1.29E-03 | 2.297 | 2.16E-02 | ++ | 578 | 723 |
| ***USP22*** | 2680086 | 0.068 | 0.053 | 1.99E-01 | 0.383 | 0.131 | 3.51E-03 | 2.296 | 2.17E-02 | ++ | 1145 | 301 |
| ***C6orf70*** | 5050021 | -0.112 | 0.056 | 4.96E-02 | -0.186 | 0.114 | 1.03E-01 | -2.292 | 2.19E-02 | -- | 206 | 1821 |
| ***EMG1*** | 4850091 | 0.153 | 0.065 | 2.02E-02 | -0.039 | 0.107 | 7.18E-01 | 2.277 | 2.28E-02 | ++ | 50 | 4119 |
| ***C4orf29*** | 5810192 | 0.130 | 0.062 | 3.80E-02 | 0.418 | 0.186 | 2.51E-02 | 2.276 | 2.29E-02 | ++ | 144 | 2474 |
| ***-*** | 5820180 | 0.075 | 0.070 | 2.84E-01 | 0.457 | 0.154 | 3.02E-03 | 2.276 | 2.29E-02 | ++ | 1883 | 147 |
| ***-*** | 3390681 | -0.186 | 0.072 | 1.09E-02 | -0.066 | 0.091 | 4.66E-01 | -2.271 | 2.31E-02 | -- | 26 | 5810 |
| ***MTERFD1*** | 4610411 | 0.102 | 0.059 | 8.92E-02 | 0.131 | 0.121 | 2.76E-01 | 2.27 | 2.32E-02 | ++ | 458 | 1048 |
| ***CAMLG*** | 6660092 | 0.116 | 0.066 | 8.02E-02 | 0.141 | 0.084 | 9.48E-02 | 2.266 | 2.35E-02 | ++ | 381 | 1221 |
| ***-*** | 1990379 | 0.052 | 0.064 | 4.17E-01 | 0.406 | 0.127 | 1.43E-03 | 2.263 | 2.36E-02 | ++ | 3140 | 73 |
| ***KARS*** | 5340291 | -0.125 | 0.059 | 3.55E-02 | -0.146 | 0.092 | 1.15E-01 | -2.256 | 2.41E-02 | -- | 120 | 2872 |
| ***-*** | 2600463 | -0.046 | 0.072 | 5.28E-01 | -0.213 | 0.117 | 6.87E-02 | -2.254 | 2.42E-02 | -- | 4199 | 38 |
| ***-*** | 2810730 | 0.035 | 0.058 | 5.44E-01 | 0.261 | 0.114 | 2.15E-02 | 2.248 | 2.46E-02 | ++ | 4268 | 37 |
| ***-*** | 7050026 | 0.083 | 0.050 | 9.96E-02 | 0.359 | 0.139 | 9.93E-03 | 2.244 | 2.48E-02 | ++ | 503 | 1057 |
| ***-*** | 7160626 | 0.093 | 0.056 | 9.93E-02 | 0.364 | 0.135 | 7.15E-03 | 2.242 | 2.50E-02 | ++ | 504 | 1062 |
| ***DPP7*** | 2140487 | -0.101 | 0.071 | 1.60E-01 | -0.224 | 0.104 | 3.18E-02 | -2.238 | 2.52E-02 | -- | 944 | 514 |
| ***RAB27A*** | 110010 | 0.110 | 0.069 | 1.13E-01 | 0.151 | 0.158 | 3.39E-01 | 2.237 | 2.53E-02 | ++ | 589 | 891 |
| ***UBE2Q1*** | 6330754 | -0.037 | 0.051 | 4.62E-01 | -0.195 | 0.100 | 5.05E-02 | -2.232 | 2.56E-02 | -- | 3521 | 64 |
| ***MBNL1*** | 5220315 | 0.079 | 0.112 | 4.80E-01 | 0.616 | 0.251 | 1.42E-02 | 2.229 | 2.58E-02 | ++ | 3665 | 61 |
| ***SPHK2*** | 3800725 | -0.099 | 0.053 | 6.75E-02 | -0.044 | 0.096 | 6.43E-01 | -2.229 | 2.58E-02 | -- | 291 | 1696 |
| ***FBXO38*** | 2320224 | 0.095 | 0.043 | 2.89E-02 | 0.188 | 0.111 | 9.04E-02 | 2.224 | 2.62E-02 | ++ | 90 | 3657 |
| ***PSMA3*** | 3460324 | 0.145 | 0.076 | 5.92E-02 | 0.114 | 0.129 | 3.74E-01 | 2.219 | 2.65E-02 | ++ | 252 | 1943 |
| ***-*** | 6660400 | 0.100 | 0.073 | 1.75E-01 | 0.097 | 0.116 | 4.04E-01 | 2.218 | 2.66E-02 | ++ | 1044 | 489 |
| ***NFATC3*** | 6510647 | -0.050 | 0.049 | 3.11E-01 | -0.196 | 0.089 | 2.77E-02 | -2.218 | 2.66E-02 | -- | 2178 | 148 |
| ***SMAD9*** | 6130609 | 0.082 | 0.043 | 6.21E-02 | 0.227 | 0.201 | 2.60E-01 | 2.217 | 2.66E-02 | ++ | 273 | 1854 |
| ***-*** | 4810048 | 0.081 | 0.080 | 3.12E-01 | 0.419 | 0.129 | 1.19E-03 | 2.215 | 2.68E-02 | ++ | 2037 | 165 |
| ***SMARCE1*** | 2650064 | 0.155 | 0.072 | 3.30E-02 | 0.153 | 0.127 | 2.28E-01 | 2.212 | 2.70E-02 | ++ | 116 | 3252 |
| ***-*** | 620280 | 0.067 | 0.069 | 3.30E-01 | 0.215 | 0.091 | 1.85E-02 | 2.211 | 2.70E-02 | ++ | 2289 | 137 |
| ***EIF4G2*** | 6220044 | 0.088 | 0.066 | 1.82E-01 | 0.109 | 0.083 | 1.87E-01 | 2.21 | 2.71E-02 | ++ | 1084 | 482 |
| ***-*** | 3370164 | -0.090 | 0.064 | 1.57E-01 | -0.351 | 0.117 | 2.82E-03 | -2.206 | 2.74E-02 | -- | 893 | 633 |
| ***C6orf190*** | 4040187 | 0.125 | 0.090 | 1.68E-01 | 0.212 | 0.186 | 2.53E-01 | 2.203 | 2.76E-02 | ++ | 992 | 566 |
| ***C11orf61*** | 7100554 | -0.100 | 0.046 | 3.03E-02 | -0.080 | 0.103 | 4.38E-01 | -2.202 | 2.77E-02 | -- | 102 | 3560 |
| ***GFM1*** | 3440195 | 0.064 | 0.065 | 3.29E-01 | 0.297 | 0.114 | 8.92E-03 | 2.2 | 2.78E-02 | ++ | 2248 | 149 |
| ***MBIP*** | 6480041 | 0.005 | 0.047 | 9.24E-01 | 0.218 | 0.084 | 9.41E-03 | 2.199 | 2.78E-02 | ++ | 8535 | 4 |
| ***-*** | 4480736 | 0.068 | 0.059 | 2.50E-01 | 0.142 | 0.100 | 1.59E-01 | 2.199 | 2.79E-02 | ++ | 1605 | 272 |
| ***-*** | 2070730 | -0.042 | 0.059 | 4.78E-01 | -0.324 | 0.105 | 2.05E-03 | -2.195 | 2.82E-02 | -- | 3701 | 69 |
| ***CARKD*** | 4060327 | -0.076 | 0.058 | 1.95E-01 | -0.250 | 0.100 | 1.22E-02 | -2.19 | 2.85E-02 | -- | 1179 | 473 |
| ***TNIP1*** | 2680100 | -0.147 | 0.109 | 1.82E-01 | -0.160 | 0.122 | 1.92E-01 | -2.187 | 2.87E-02 | -- | 1109 | 520 |
| ***YIPF4*** | 7320379 | 0.099 | 0.074 | 1.81E-01 | 0.360 | 0.101 | 3.45E-04 | 2.187 | 2.87E-02 | ++ | 1081 | 548 |
| ***FXR2*** | 5690280 | -0.099 | 0.064 | 1.25E-01 | -0.233 | 0.110 | 3.46E-02 | -2.187 | 2.88E-02 | -- | 664 | 930 |
| ***NDRG2*** | 4210411 | -0.079 | 0.050 | 1.14E-01 | -0.413 | 0.136 | 2.35E-03 | -2.182 | 2.91E-02 | -- | 654 | 965 |
| ***GBGT1*** | 4490687 | -0.131 | 0.087 | 1.37E-01 | -0.053 | 0.155 | 7.31E-01 | -2.181 | 2.92E-02 | -- | 761 | 817 |
| ***CD74*** | 7040008 | 0.147 | 0.093 | 1.16E-01 | 0.123 | 0.144 | 3.93E-01 | 2.179 | 2.93E-02 | ++ | 622 | 1023 |
| ***CNPY4*** | 6200743 | 0.068 | 0.044 | 1.26E-01 | -0.003 | 0.101 | 9.79E-01 | 2.175 | 2.97E-02 | ++ | 641 | 1004 |
| ***CSF2RB*** | 3450349 | -0.087 | 0.060 | 1.52E-01 | -0.086 | 0.103 | 4.08E-01 | -2.173 | 2.98E-02 | -- | 884 | 728 |
| ***-*** | 6770494 | 0.095 | 0.054 | 7.75E-02 | -0.019 | 0.099 | 8.47E-01 | 2.168 | 3.01E-02 | ++ | 336 | 1833 |
| ***SNAI3*** | 4880017 | -0.046 | 0.052 | 3.78E-01 | -0.216 | 0.122 | 7.53E-02 | -2.167 | 3.03E-02 | -- | 2732 | 124 |
| ***DOCK11*** | 7610730 | 0.148 | 0.093 | 1.15E-01 | 0.426 | 0.256 | 9.71E-02 | 2.166 | 3.03E-02 | ++ | 579 | 1131 |
| ***PXK*** | 6250458 | -0.052 | 0.044 | 2.47E-01 | -0.254 | 0.079 | 1.37E-03 | -2.164 | 3.05E-02 | -- | 1655 | 305 |
| ***-*** | 3850600 | 0.117 | 0.052 | 2.75E-02 | 0.006 | 0.119 | 9.62E-01 | 2.162 | 3.06E-02 | ++ | 89 | 4237 |
| ***CWF19L1*** | 240097 | -0.108 | 0.055 | 5.05E-02 | -0.048 | 0.095 | 6.14E-01 | -2.16 | 3.08E-02 | -- | 200 | 2708 |
| ***SIT1*** | 4280053 | -0.142 | 0.065 | 3.02E-02 | -0.177 | 0.135 | 1.92E-01 | -2.159 | 3.08E-02 | -- | 107 | 3923 |
| ***-*** | 1510072 | -0.095 | 0.063 | 1.34E-01 | -0.230 | 0.100 | 2.12E-02 | -2.154 | 3.12E-02 | -- | 715 | 954 |
| ***ZNF473*** | 3360735 | -0.082 | 0.075 | 2.73E-01 | -0.195 | 0.074 | 7.95E-03 | -2.152 | 3.14E-02 | -- | 1793 | 281 |
| ***PDCD5*** | 1240685 | -0.083 | 0.069 | 2.28E-01 | -0.079 | 0.090 | 3.82E-01 | -2.149 | 3.16E-02 | -- | 1490 | 381 |
| ***RAB27A*** | 2680044 | 0.149 | 0.074 | 4.69E-02 | 0.266 | 0.149 | 7.45E-02 | 2.149 | 3.16E-02 | ++ | 181 | 2948 |
| ***SPATA20*** | 5050520 | -0.152 | 0.093 | 1.06E-01 | 0.205 | 0.295 | 4.88E-01 | -2.149 | 3.16E-02 | -- | 558 | 1251 |
| ***PUS7*** | 3940615 | -0.121 | 0.059 | 4.26E-02 | -0.216 | 0.102 | 3.30E-02 | -2.141 | 3.23E-02 | -- | 165 | 3261 |
| ***-*** | 5080291 | 0.085 | 0.058 | 1.42E-01 | 0.058 | 0.089 | 5.13E-01 | 2.14 | 3.24E-02 | ++ | 755 | 949 |
| ***-*** | 3520753 | -0.141 | 0.076 | 6.47E-02 | -0.016 | 0.089 | 8.55E-01 | -2.134 | 3.28E-02 | -- | 289 | 2226 |
| ***SEC62*** | 110327 | 0.088 | 0.041 | 3.32E-02 | 0.119 | 0.194 | 5.38E-01 | 2.129 | 3.32E-02 | ++ | 109 | 4162 |
| ***HLA-G*** | 2070088 | 0.278 | 0.168 | 1.02E-01 | 0.163 | 0.173 | 3.45E-01 | 2.128 | 3.33E-02 | ++ | 527 | 1447 |
| ***CES2*** | 3520372 | 0.074 | 0.062 | 2.31E-01 | 0.158 | 0.088 | 7.11E-02 | 2.126 | 3.35E-02 | ++ | 1509 | 413 |
| ***ZNF784*** | 4590347 | 0.085 | 0.052 | 1.03E-01 | 0.160 | 0.097 | 9.72E-02 | 2.126 | 3.35E-02 | ++ | 544 | 1396 |
| ***AL353715.21\STMN3*** | 6480246 | -0.161 | 0.066 | 1.69E-02 | -0.035 | 0.148 | 8.15E-01 | -2.125 | 3.36E-02 | -- | 45 | 5876 |
| ***TRMT61A*** | 4150484 | -0.088 | 0.051 | 8.61E-02 | 0.006 | 0.110 | 9.57E-01 | -2.124 | 3.36E-02 | -- | 437 | 1722 |
| ***-*** | 3450743 | -0.035 | 0.058 | 5.49E-01 | -0.213 | 0.089 | 1.66E-02 | -2.123 | 3.37E-02 | -- | 4428 | 66 |
| ***CORO7*** | 3360167 | -0.099 | 0.070 | 1.61E-01 | -0.252 | 0.142 | 7.57E-02 | -2.121 | 3.39E-02 | -- | 913 | 847 |
| ***SF3B3*** | 1090239 | -0.079 | 0.063 | 2.10E-01 | -0.149 | 0.089 | 9.32E-02 | -2.121 | 3.39E-02 | -- | 1336 | 534 |
| ***-*** | 3800670 | -0.089 | 0.050 | 7.84E-02 | 0.036 | 0.112 | 7.47E-01 | -2.119 | 3.41E-02 | -- | 357 | 2014 |
| ***-*** | 3450672 | -0.096 | 0.054 | 7.95E-02 | -0.002 | 0.084 | 9.83E-01 | -2.115 | 3.45E-02 | -- | 373 | 1956 |
| ***C14orf10*** | 1820072 | 0.056 | 0.086 | 5.22E-01 | 0.470 | 0.168 | 5.10E-03 | 2.114 | 3.46E-02 | ++ | 4128 | 82 |
| ***DIABLO*** | 4890717 | -0.043 | 0.048 | 3.68E-01 | -0.157 | 0.072 | 2.81E-02 | -2.113 | 3.46E-02 | -- | 2592 | 173 |
| ***MEF2C*** | 4040162 | 0.123 | 0.090 | 1.76E-01 | 0.405 | 0.197 | 3.99E-02 | 2.113 | 3.46E-02 | ++ | 996 | 786 |
| ***-*** | 2070440 | 0.076 | 0.056 | 1.79E-01 | 0.095 | 0.083 | 2.56E-01 | 2.113 | 3.46E-02 | ++ | 978 | 804 |
| ***PRCP*** | 6110386 | 0.137 | 0.066 | 4.14E-02 | 0.112 | 0.158 | 4.79E-01 | 2.112 | 3.47E-02 | ++ | 166 | 3503 |
| ***ABHD12*** | 7100615 | -0.028 | 0.051 | 5.84E-01 | -0.304 | 0.124 | 1.43E-02 | -2.112 | 3.47E-02 | -- | 5068 | 52 |
| ***FBXL10*** | 1190246 | 0.073 | 0.063 | 2.51E-01 | 0.172 | 0.087 | 4.89E-02 | 2.111 | 3.47E-02 | ++ | 1664 | 378 |
| ***AC134878.3-1*** | 6760343 | 0.182 | 0.094 | 5.48E-02 | 0.450 | 0.285 | 1.14E-01 | 2.111 | 3.48E-02 | ++ | 213 | 2924 |
| ***C17orf70*** | 4540064 | -0.119 | 0.064 | 6.60E-02 | -0.087 | 0.118 | 4.62E-01 | -2.109 | 3.50E-02 | -- | 287 | 2421 |
| ***-*** | 1230196 | 0.087 | 0.078 | 2.68E-01 | 0.321 | 0.185 | 8.23E-02 | 2.109 | 3.50E-02 | ++ | 1769 | 346 |
| ***RBM22*** | 6200630 | 0.048 | 0.052 | 3.58E-01 | 0.109 | 0.057 | 5.69E-02 | 2.108 | 3.50E-02 | ++ | 2567 | 180 |
| ***TUBA1B*** | 6510176 | 0.043 | 0.086 | 6.19E-01 | 0.227 | 0.090 | 1.15E-02 | 2.107 | 3.51E-02 | ++ | 5160 | 51 |
| ***FCER1A\OR10J5*** | 3360615 | -0.114 | 0.182 | 5.34E-01 | -0.590 | 0.277 | 3.29E-02 | -2.105 | 3.53E-02 | -- | 4243 | 80 |
| ***OAS1*** | 3450180 | -0.175 | 0.135 | 1.98E-01 | -0.363 | 0.426 | 3.94E-01 | -2.102 | 3.56E-02 | -- | 1211 | 656 |
| ***AGGF1*** | 70239 | 0.121 | 0.059 | 4.28E-02 | 0.079 | 0.182 | 6.65E-01 | 2.101 | 3.56E-02 | ++ | 170 | 3537 |
| ***-*** | 4230437 | -0.130 | 0.062 | 3.76E-02 | -0.134 | 0.103 | 1.93E-01 | -2.099 | 3.58E-02 | -- | 134 | 3990 |
| ***ARF1*** | 1430259 | -0.108 | 0.065 | 9.86E-02 | -0.206 | 0.106 | 5.22E-02 | -2.097 | 3.60E-02 | -- | 518 | 1646 |
| ***-*** | 4220241 | -0.056 | 0.043 | 1.94E-01 | -0.193 | 0.099 | 5.15E-02 | -2.093 | 3.63E-02 | -- | 1227 | 663 |
| ***NCOA1*** | 160411 | 0.067 | 0.085 | 4.33E-01 | 0.276 | 0.106 | 9.34E-03 | 2.082 | 3.73E-02 | ++ | 3181 | 133 |
| ***TAP2*** | 3940477 | 0.062 | 0.096 | 5.20E-01 | 0.253 | 0.154 | 1.01E-01 | 2.081 | 3.74E-02 | ++ | 4166 | 89 |
| ***NR1D2*** | 2640707 | 0.104 | 0.064 | 1.07E-01 | 0.343 | 0.182 | 5.95E-02 | 2.08 | 3.75E-02 | ++ | 542 | 1639 |
| ***-*** | 3850112 | -0.015 | 0.081 | 8.54E-01 | -0.253 | 0.203 | 2.12E-01 | -2.079 | 3.76E-02 | -- | 7738 | 17 |
| ***SUV420H1*** | 3850343 | 0.071 | 0.051 | 1.66E-01 | 0.180 | 0.103 | 8.05E-02 | 2.078 | 3.77E-02 | ++ | 908 | 984 |
| ***CLPX*** | 6760050 | 0.011 | 0.062 | 8.55E-01 | 0.290 | 0.115 | 1.20E-02 | 2.076 | 3.79E-02 | ++ | 7804 | 16 |
| ***-*** | 7510470 | 0.157 | 0.091 | 8.71E-02 | 0.502 | 0.461 | 2.76E-01 | 2.075 | 3.80E-02 | ++ | 410 | 2041 |
| ***PISD*** | 1440278 | -0.119 | 0.093 | 2.02E-01 | -0.207 | 0.127 | 1.02E-01 | -2.073 | 3.82E-02 | -- | 1245 | 698 |
| ***C1orf212*** | 7040411 | 0.105 | 0.057 | 6.75E-02 | -0.001 | 0.079 | 9.95E-01 | 2.073 | 3.82E-02 | ++ | 285 | 2695 |
| ***ING3*** | 7550707 | 0.014 | 0.093 | 8.77E-01 | 0.141 | 0.148 | 3.42E-01 | 2.073 | 3.82E-02 | ++ | 7975 | 13 |
| ***-*** | 5090647 | 0.131 | 0.051 | 1.13E-02 | 0.112 | 0.083 | 1.76E-01 | 2.071 | 3.83E-02 | ++ | 29 | 7881 |
| ***-*** | 940373 | -0.012 | 0.064 | 8.55E-01 | -0.361 | 0.113 | 1.37E-03 | -2.071 | 3.83E-02 | -- | 7952 | 14 |
| ***CACNA2D2*** | 3120180 | 0.135 | 0.053 | 1.27E-02 | 0.083 | 0.170 | 6.24E-01 | 2.069 | 3.85E-02 | ++ | 32 | 7620 |
| ***SPG11*** | 2100524 | 0.081 | 0.071 | 2.55E-01 | 0.367 | 0.135 | 6.46E-03 | 2.06 | 3.94E-02 | ++ | 1716 | 464 |
| ***DHRS9*** | 630315 | 0.198 | 0.171 | 2.51E-01 | 0.479 | 0.232 | 3.90E-02 | 2.058 | 3.95E-02 | ++ | 1608 | 509 |
| ***TESC*** | 5050681 | -0.251 | 0.183 | 1.72E-01 | -0.227 | 0.162 | 1.60E-01 | -2.056 | 3.98E-02 | -- | 1005 | 953 |
| ***-*** | 4560047 | 0.204 | 0.104 | 5.10E-02 | 0.025 | 0.179 | 8.88E-01 | 2.055 | 3.99E-02 | ++ | 207 | 3422 |
| ***-*** | 5560349 | 0.056 | 0.054 | 2.98E-01 | 0.069 | 0.057 | 2.22E-01 | 2.055 | 3.99E-02 | ++ | 2000 | 350 |
| ***PLEK*** | 4810020 | 0.123 | 0.089 | 1.67E-01 | 0.247 | 0.123 | 4.43E-02 | 2.052 | 4.02E-02 | ++ | 975 | 997 |
| ***ACIN1*** | 4860204 | -0.082 | 0.050 | 1.03E-01 | -0.150 | 0.099 | 1.32E-01 | -2.051 | 4.03E-02 | -- | 525 | 1855 |
| ***-*** | 1580309 | -0.142 | 0.127 | 2.66E-01 | -0.221 | 0.101 | 2.80E-02 | -2.05 | 4.04E-02 | -- | 1743 | 471 |
| ***C9orf80*** | 990475 | 0.147 | 0.059 | 1.39E-02 | 0.231 | 0.119 | 5.18E-02 | 2.047 | 4.07E-02 | ++ | 34 | 7636 |
| ***PPOX*** | 3120609 | -0.092 | 0.057 | 1.13E-01 | -0.101 | 0.088 | 2.48E-01 | -2.047 | 4.07E-02 | -- | 606 | 1592 |
| ***PSME1*** | 150047 | 0.144 | 0.066 | 3.18E-02 | 0.030 | 0.089 | 7.33E-01 | 2.045 | 4.09E-02 | ++ | 112 | 4851 |
| ***GPS1*** | 1570113 | 0.096 | 0.051 | 6.45E-02 | 0.068 | 0.076 | 3.75E-01 | 2.044 | 4.09E-02 | ++ | 240 | 3188 |
| ***EEF2*** | 1580292 | -0.178 | 0.090 | 5.07E-02 | -0.101 | 0.109 | 3.56E-01 | -2.044 | 4.10E-02 | -- | 205 | 3525 |
| ***C12orf32*** | 3180553 | 0.048 | 0.052 | 3.57E-01 | 0.046 | 0.095 | 6.27E-01 | 2.043 | 4.11E-02 | ++ | 2434 | 263 |
| ***-*** | 940288 | 0.051 | 0.102 | 6.21E-01 | 0.547 | 0.202 | 6.72E-03 | 2.042 | 4.11E-02 | ++ | 5061 | 70 |
| ***ZNF586*** | 1050626 | 0.018 | 0.045 | 6.92E-01 | 0.262 | 0.123 | 3.35E-02 | 2.039 | 4.14E-02 | ++ | 5733 | 53 |
| ***ATG5*** | 2030228 | 0.072 | 0.049 | 1.44E-01 | 0.064 | 0.107 | 5.48E-01 | 2.037 | 4.16E-02 | ++ | 817 | 1232 |
| ***PJA2*** | 520431 | 0.100 | 0.122 | 4.16E-01 | 0.554 | 0.275 | 4.41E-02 | 2.035 | 4.19E-02 | ++ | 3041 | 181 |
| ***-*** | 3190288 | -0.338 | 0.151 | 2.70E-02 | -0.065 | 0.129 | 6.16E-01 | -2.032 | 4.22E-02 | -- | 92 | 5455 |
| ***SFRS2\C17orf95*** | 1030440 | -0.083 | 0.046 | 7.15E-02 | -0.206 | 0.093 | 2.71E-02 | -2.03 | 4.24E-02 | -- | 329 | 2729 |
| ***RFNG*** | 270204 | -0.098 | 0.045 | 3.16E-02 | -0.153 | 0.094 | 1.05E-01 | -2.029 | 4.25E-02 | -- | 117 | 4924 |
| ***C16orf53*** | 4230066 | -0.058 | 0.058 | 3.24E-01 | -0.362 | 0.133 | 6.46E-03 | -2.028 | 4.26E-02 | -- | 2234 | 324 |
| ***CLEC11A*** | 1940048 | 0.056 | 0.057 | 3.29E-01 | 0.378 | 0.191 | 4.80E-02 | 2.02 | 4.34E-02 | ++ | 2175 | 355 |
| ***-*** | 3610192 | -0.172 | 0.095 | 7.40E-02 | 0.145 | 0.221 | 5.13E-01 | -2.019 | 4.35E-02 | -- | 327 | 2833 |
| ***RPL32P3*** | 4200551 | 0.118 | 0.053 | 2.75E-02 | 0.328 | 0.198 | 9.71E-02 | 2.017 | 4.37E-02 | ++ | 86 | 5724 |
| ***SLC25A3*** | 4040224 | 0.093 | 0.061 | 1.33E-01 | 0.152 | 0.100 | 1.27E-01 | 2.016 | 4.38E-02 | ++ | 721 | 1475 |
| ***PRDX5*** | 520750 | -0.011 | 0.117 | 9.27E-01 | -0.264 | 0.095 | 5.65E-03 | -2.014 | 4.40E-02 | -- | 8738 | 12 |
| ***ETFDH\C4orf46*** | 580609 | 0.104 | 0.052 | 4.65E-02 | 0.145 | 0.115 | 2.06E-01 | 2.013 | 4.41E-02 | ++ | 179 | 4233 |
| ***-*** | 7210487 | -0.089 | 0.056 | 1.14E-01 | -0.053 | 0.104 | 6.07E-01 | -2.012 | 4.42E-02 | -- | 614 | 1745 |
| ***S100A10*** | 540681 | 0.078 | 0.079 | 3.21E-01 | 0.292 | 0.105 | 5.32E-03 | 2.01 | 4.44E-02 | ++ | 2221 | 357 |
| ***MMADHC*** | 4180196 | 0.044 | 0.062 | 4.83E-01 | 0.257 | 0.104 | 1.36E-02 | 2.01 | 4.45E-02 | ++ | 3724 | 135 |
| ***PTPN1*** | 4390703 | -0.083 | 0.071 | 2.45E-01 | -0.163 | 0.074 | 2.73E-02 | -2.009 | 4.45E-02 | -- | 1567 | 658 |
| ***CCNYL1*** | 1410398 | 0.083 | 0.049 | 9.35E-02 | 0.202 | 0.092 | 2.84E-02 | 2.008 | 4.47E-02 | ++ | 433 | 2413 |
| ***ITCH*** | 7560538 | 0.063 | 0.047 | 1.84E-01 | 0.253 | 0.166 | 1.28E-01 | 2.005 | 4.50E-02 | ++ | 1071 | 1056 |
| ***SERINC3*** | 7380139 | -0.082 | 0.073 | 2.62E-01 | -0.185 | 0.116 | 1.10E-01 | -2.002 | 4.52E-02 | -- | 1771 | 560 |
| ***-*** | 7160327 | 0.146 | 0.060 | 1.63E-02 | 0.151 | 0.098 | 1.25E-01 | 2.002 | 4.53E-02 | ++ | 40 | 7511 |
| ***EIF3S1*** | 4280020 | 0.048 | 0.052 | 3.53E-01 | 0.297 | 0.124 | 1.70E-02 | 2.001 | 4.54E-02 | ++ | 2379 | 331 |
| ***ACSL3*** | 1580524 | 0.089 | 0.066 | 1.79E-01 | 0.242 | 0.116 | 3.70E-02 | 1.995 | 4.61E-02 | ++ | 1056 | 1096 |
| ***-*** | 780068 | 0.108 | 0.063 | 8.72E-02 | 0.043 | 0.130 | 7.38E-01 | 1.994 | 4.61E-02 | ++ | 403 | 2589 |
| ***INTS12*** | 4860553 | 0.065 | 0.049 | 1.90E-01 | 0.068 | 0.071 | 3.34E-01 | 1.993 | 4.62E-02 | ++ | 1106 | 1053 |
| ***MTX2*** | 460072 | 0.057 | 0.063 | 3.65E-01 | 0.068 | 0.094 | 4.71E-01 | 1.992 | 4.64E-02 | ++ | 2556 | 304 |
| ***-*** | 510672 | 0.097 | 0.059 | 1.03E-01 | 0.139 | 0.094 | 1.40E-01 | 1.991 | 4.65E-02 | ++ | 529 | 2168 |
| ***-*** | 6380537 | -0.091 | 0.055 | 9.87E-02 | -0.094 | 0.095 | 3.24E-01 | -1.99 | 4.66E-02 | -- | 510 | 2271 |
| ***EVI2B\NF1*** | 7050152 | 0.028 | 0.078 | 7.18E-01 | 0.522 | 0.200 | 8.91E-03 | 1.987 | 4.69E-02 | ++ | 6163 | 58 |
| ***SEMA4F*** | 3060754 | -0.079 | 0.047 | 9.62E-02 | -0.101 | 0.098 | 3.07E-01 | -1.986 | 4.71E-02 | -- | 553 | 2096 |
| ***-*** | 1440348 | -0.114 | 0.079 | 1.53E-01 | -0.157 | 0.138 | 2.53E-01 | -1.985 | 4.72E-02 | -- | 885 | 1350 |
| ***MRPS6*** | 6400195 | 0.157 | 0.065 | 1.75E-02 | -0.001 | 0.100 | 9.92E-01 | 1.984 | 4.73E-02 | ++ | 43 | 7443 |
| ***CHD1L*** | 5870148 | -0.087 | 0.056 | 1.21E-01 | -0.219 | 0.100 | 2.86E-02 | -1.983 | 4.73E-02 | -- | 704 | 1679 |
| ***CYFIP1*** | 6420239 | -0.129 | 0.058 | 2.96E-02 | 0.000 | 0.146 | 9.97E-01 | -1.983 | 4.74E-02 | -- | 103 | 5680 |
| ***FAM172A*** | 4860379 | 0.108 | 0.052 | 3.96E-02 | 0.049 | 0.098 | 6.18E-01 | 1.981 | 4.76E-02 | ++ | 145 | 4937 |
| ***-*** | 990273 | -0.155 | 0.083 | 6.56E-02 | -0.124 | 0.201 | 5.35E-01 | -1.977 | 4.80E-02 | -- | 284 | 3409 |
| ***-*** | 4610390 | 0.047 | 0.058 | 4.23E-01 | 0.081 | 0.056 | 1.52E-01 | 1.973 | 4.85E-02 | ++ | 3137 | 227 |
| ***LSM14A*** | 2340328 | 0.106 | 0.064 | 1.00E-01 | 0.207 | 0.132 | 1.17E-01 | 1.971 | 4.87E-02 | ++ | 514 | 2374 |
| ***FURIN*** | 3870706 | -0.030 | 0.089 | 7.36E-01 | -0.353 | 0.139 | 1.12E-02 | -1.971 | 4.87E-02 | -- | 6349 | 57 |
| ***RPS6KA5*** | 6220438 | 0.040 | 0.093 | 6.66E-01 | 0.366 | 0.145 | 1.14E-02 | 1.971 | 4.88E-02 | ++ | 5501 | 81 |
| ***DPP7*** | 670608 | -0.101 | 0.059 | 8.70E-02 | -0.130 | 0.105 | 2.19E-01 | -1.97 | 4.89E-02 | -- | 439 | 2668 |
| ***GTF3A*** | 270619 | 0.121 | 0.068 | 7.78E-02 | 0.096 | 0.102 | 3.47E-01 | 1.968 | 4.91E-02 | ++ | 377 | 2914 |
| ***NAALADL1*** | 4120626 | -0.063 | 0.058 | 2.77E-01 | -0.270 | 0.121 | 2.58E-02 | -1.966 | 4.93E-02 | -- | 1873 | 580 |
| ***-*** | 3460491 | -0.093 | 0.055 | 9.05E-02 | -0.262 | 0.134 | 5.04E-02 | -1.966 | 4.93E-02 | -- | 492 | 2507 |
| ***C11orf73*** | 780440 | -0.004 | 0.059 | 9.47E-01 | -0.368 | 0.090 | 4.08E-05 | -1.964 | 4.96E-02 | -- | 8965 | 18 |
| ***AC073517.6*** | 1710746 | -0.064 | 0.052 | 2.23E-01 | -0.144 | 0.080 | 7.02E-02 | -1.963 | 4.96E-02 | -- | 1376 | 912 |
| ***NUMA1*** | 4120338 | -0.046 | 0.046 | 3.13E-01 | -0.355 | 0.114 | 1.92E-03 | -1.963 | 4.96E-02 | -- | 2258 | 433 |
| ***SEC24B*** | 7000019 | 0.053 | 0.049 | 2.86E-01 | 0.280 | 0.129 | 3.01E-02 | 1.963 | 4.97E-02 | ++ | 1860 | 590 |
| ***CTBS\SPATA1*** | 2850184 | 0.076 | 0.073 | 3.01E-01 | 0.390 | 0.149 | 9.04E-03 | 1.96 | 4.99E-02 | ++ | 1937 | 568 |

**SUPPLEMENTAL FIGURES**

**Supplementary Figure 1:** Gene expression levels of *C1orf38* versus the joint effusion grades. **A**) Boxplots for RS. **B**) Boxplots for GARP cases with knee OA. **C**) Boxplots for GARP cases without knee OA.

**A.**

**
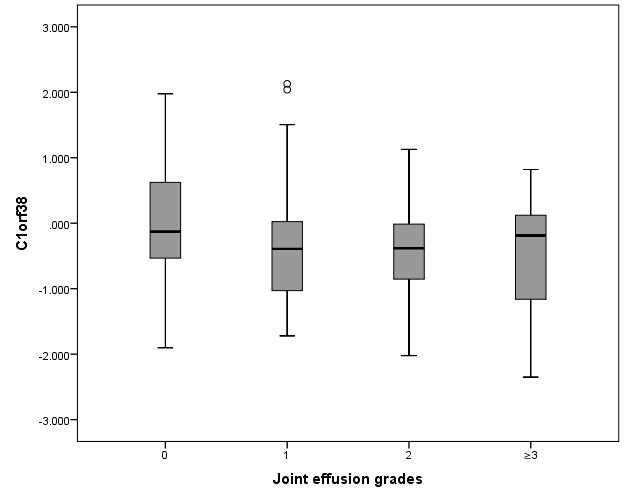
**

**B.**

**
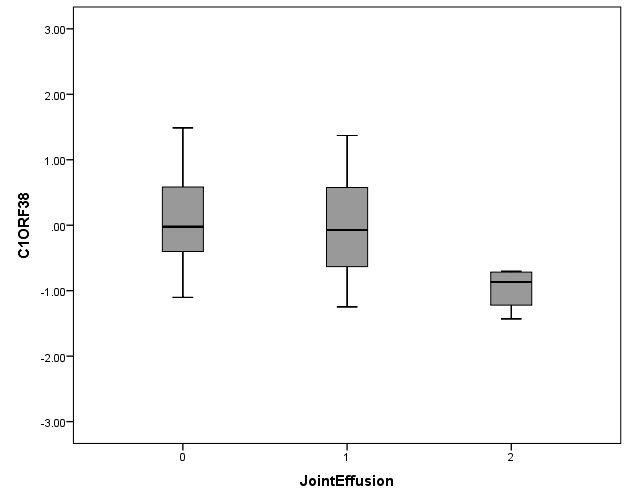
**

**C.**

**
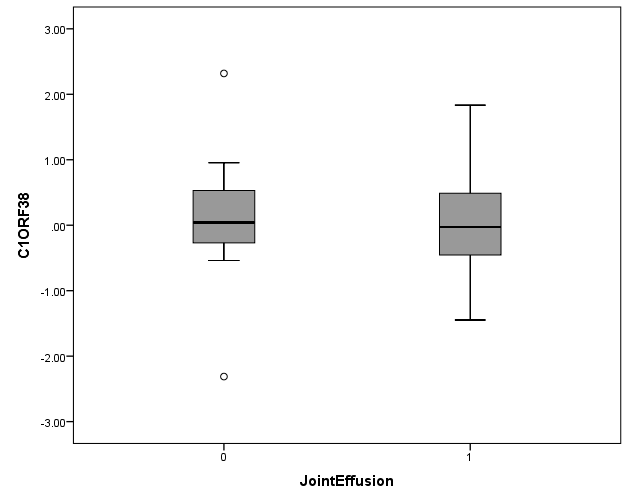
**

**Supplementary Figure 2:** Gene expression levels of *DYNLL2* versus the joint effusion grades. **A**) Boxplots for RS. **B**) Boxplots for GARP cases with knee OA. **C**) Boxplots for GARP cases without knee OA.

**A.**

**
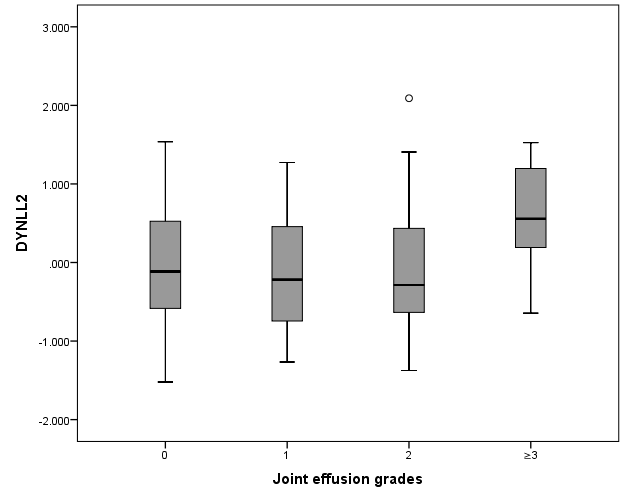
**

**B.**

**
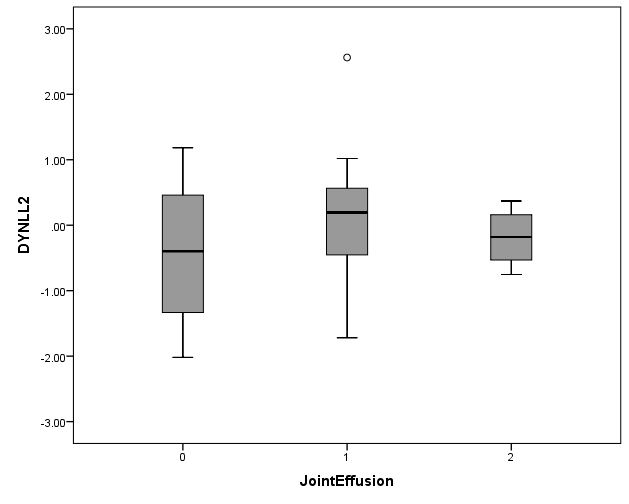
**

**C.**

**
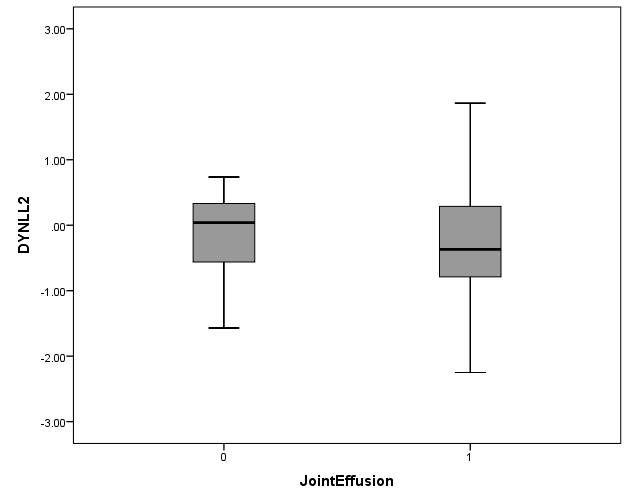
**

**Supplementary Figure 3:** Gene expression levels of *NFATC1* versus the joint effusion grades. **A**) Boxplots for RS. **B**) Boxplots for GARP cases with knee OA. **C**) Boxplots for GARP cases without knee OA.

**A.**

**
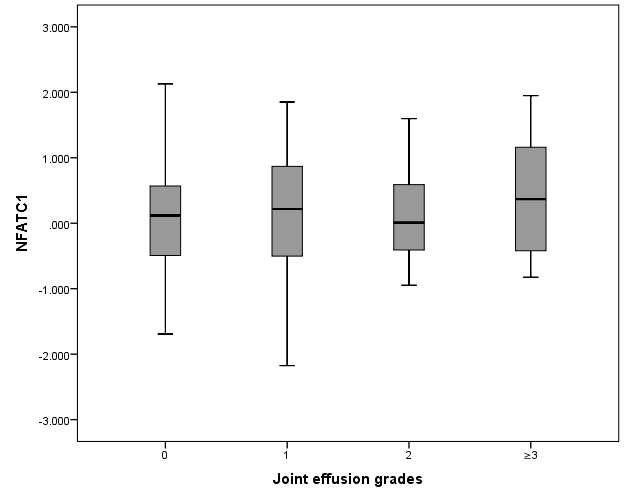
**

**B.**

**
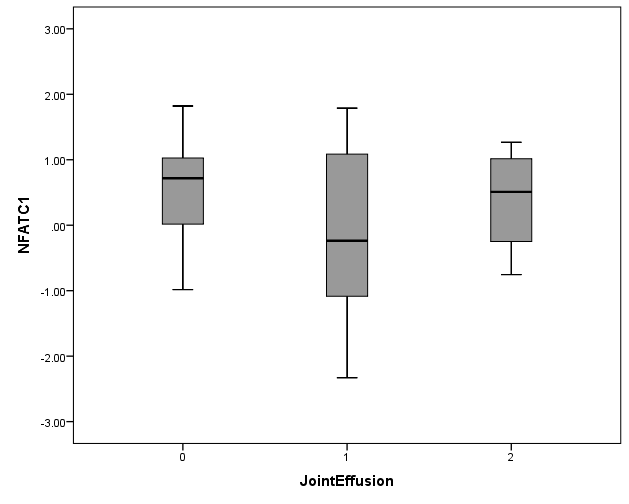
**

**C.**


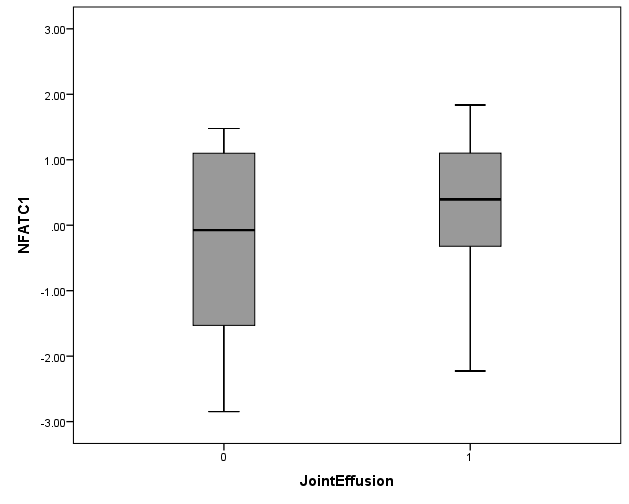


**Supplementary Figure 4:** Gene expression levels of *RBM4* versus the joint effusion grades. **A**) Boxplots for RS. **B**) Boxplots for GARP cases with knee OA. **C**) Boxplots for GARP cases without knee OA.

**A.**

**
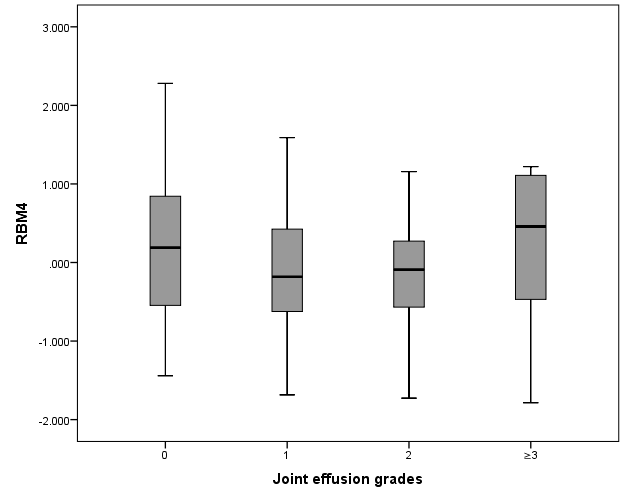
**

**B.**


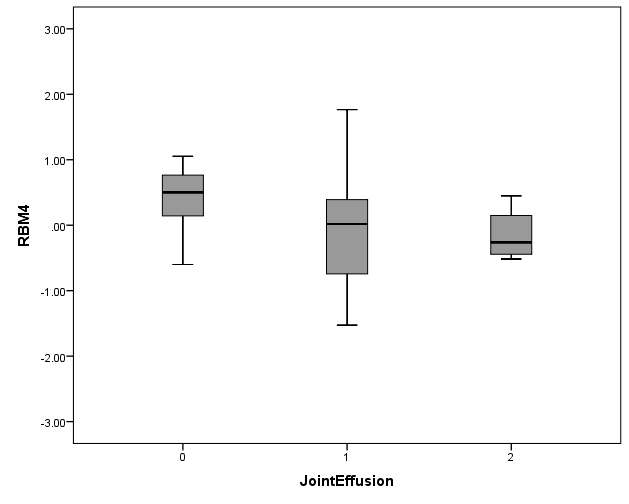


**C.**

**
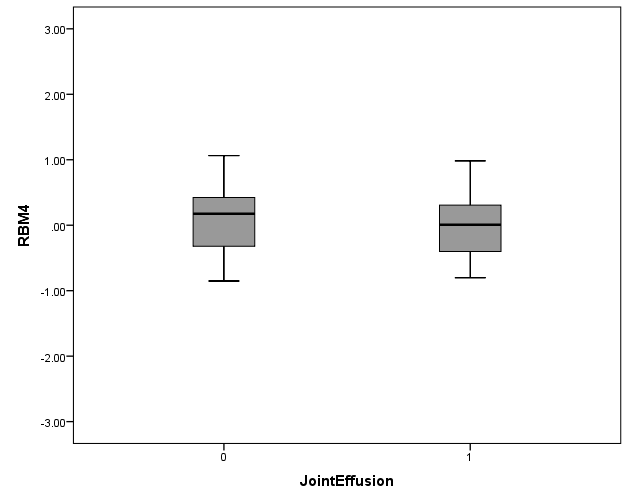
**
