# Supplementary material for: Associations between joint effusion in the knee and gene expression levels in the circulation: a meta-analysis
Source: F1000Res. 2016 Jan 27;5:109. [Version 1] doi: 10.12688/f1000research.7763.1 (PMC4837985; doi:10.12688/f1000research.7763.1)
Supplement: Peer review history

# Peer review: Referee response for version 1

**DOI:** 10.5256/f1000research.8357.r13227

**Published:** 2016-04-18

**Authors:** Gauthaman Kalamegam

**Affiliations:** 1 Center of Excellence in Genomic Medicine Research (CEGMR), King Abdulaziz University, Jeddah, Saudi Arabia

This  study investigates the associations between joint effusion in the Knee and gene expression levels in the circulation. This is a well performed study that had led to the identification of 189 unique genes in blood  with joint effusion in knee and few candidate biomarkers. Although the sample size was less and had cohort heterogeneity the authors have made a very systematic approach to identify  genes and biological pathways involved in osteoarthritis.

I have read this submission. I believe that I have an appropriate level of expertise to confirm that it is of an acceptable scientific standard.

# Peer review: Referee response for version 1

**DOI:** 10.5256/f1000research.8357.r13226

**Published:** 2016-04-18

**Authors:** Csaba Matta

**Affiliations:** 1 Department of Veterinary Preclinical Sciences, University of Surrey, Guilford, UK

Identifying and validating biomarkers from easily accessible body fluids such as peripheral blood for early (pre-radiographic) detection of osteoarthritis (OA), or at least OA susceptibility, are of key importance in the field. The authors of this manuscript aimed to analyse gene expression levels associated with joint effusion in two cohorts of patients either in the Genetics, Arthrosis and Progression study (GARP) or in the Rotterdam study (RS). 

This is a very well presented and conducted study, with results that may hopefully advance the field.

In my opinion, the major limitation of this study (which is actually stated and recognised by the authors as well) is that the two cohorts on which the study was performed are very different in nature; GARP is a cohort of clinical OA cases, whilst RS is a population-based study. This might be the reason as to why the top 5 genes in the two cohorts were different. Perhaps it would be useful to check whether the top genes in the GARP study (in which more than 50% of the subjects had OA) have any predictive value for pre-radiographic OA in the RS study. This question could of course only be answered over time.

Following on the comment of the first referee regarding the inflammatory/non-inflammatory OA phenotypes, it would also be interesting to see whether the same GO pathways would be identified if patients with inflammatory/non-inflammatory OA were separately analysed.

I have read this submission. I believe that I have an appropriate level of expertise to confirm that it is of an acceptable scientific standard.

# Peer review: Referee response for version 1

**DOI:** 10.5256/f1000research.8357.r13228

**Published:** 2016-04-15

**Authors:** Richard Barrett-Jolley

**Affiliations:** 1 Department of Musculoskeletal Biology, University of Liverpool, Liverpool, UK

This is an interesting and well conducted study that addresses a very important, and topical question.  The discovery of a biomarker of osteoarthritis in the blood would be somewhat of a Holy Grail in the field and so it is important that investigators look for suitable correlations within datasets such as the Rotterdam Study. I have no major concerns with this study, but have a number of comments and also some small points that the authors may be able to clarify.

1. A practical limitation of studies such as these, comparing biomarker level data from patients with higher levels of joint effusion already, is that OA may have already progressed to a point where a biomarker is no longer so useful. That said, on its discovery, such a biomarker would then of course be a good candidate to analyse at earlier stages of OA to then determine if it is a useful OA predictor prior to MRI-level changes. I was curious to know, if long term, these authors will be able to correlate expression levels of genes expressed in blood of healthy people which then end up with OA at a later stage?
2. It has emerged over recent years that “osteoarthritis” probably represents a family of conditions, some of which have a strong component of inflammation and some which do not. I was not sure that the distinction between inflammation being an important contributor to all OA or just some subtypes of OA was clearly made in the introduction. My understanding is that it is the latter.
3. The authors comment that this is a small sample size at some point. I was not really sure it was. I realise that for some GWAS, 1000s of patients are necessary to detect small odds ratios; however, I thought that if a biomarker was to be useful diagnostically, the change would have to be substantial enough that it should manifest statistically significant differences, even in just a few patients. Therefore, I felt, in the context of this study, it was an unnecessary self-criticism.
4. I applaud the authors for already getting the new data in the GEO database, some groups take many years to upload this, which can be frustrating. I did miss, initially that this meta-analysis pertained to expression datasets uploaded in May and July 2013. Somehow (I think it was reference to RNA extraction methods), at first, I thought this had been newly acquired. Would the words “pre-existing datasets” be useful in the methods, or would most people consider the word “met-analysis” in the title means all the data is pre-existing?
5. The definition of “significant” looked a little arbitrary to me. I know p<=0.05 is arbitrary in itself, but it has been a standard for many years now. But 0.05 in > 50% of samples seemed strange to me. The authors state that the overall FDR is very small, but what is it? Can it be calculated or estimated? I think the “(we won’t get false positive genes)” could be deleted? Benjamini Hochberg correction is used later in the pathway analysis, but not here in the regression/differential gene detection section. Is there a good reason for this?
6. In the differential expression section (not the regression) “Effect Size” is not being used in the normal context I use it. Does it mean “ratio”?
7. Table 2; the key table in the study. I felt the legend could be more helpful, and include more detail?
8. Table 2: “Top genes”? “top” in what way? Lowest p-val? Largest change?
9. My feeling is that the pathway analysis basically revealed no significantly enriched pathways, once you excluded multiple comparison error? I thought that was quite interesting (and negative data is of course valuable), but if I am correct in my interpretation, this is most certainly not stated clearly in the abstract or elsewhere.

I have read this submission. I believe that I have an appropriate level of expertise to confirm that it is of an acceptable scientific standard.
